# Metagenomic Analysis of Ampelographic Collections of Dagestan Revealed the Presence of Two Novel Grapevine Viruses

**DOI:** 10.3390/v14122623

**Published:** 2022-11-24

**Authors:** Darya Shvets, Kirill Sandomirsky, Elena Porotikova, Svetlana Vinogradova

**Affiliations:** Skryabin Institute of Bioengineering, Research Center of Biotechnology of the Russian Academy of Sciences, Leninsky Prospect, 33, Build. 2, 119071 Moscow, Russia

**Keywords:** *Vitis vinifera*, virome, RNA-seq, diagnostics, novel grapevine viruses, grapevine umbra-like virus, dsDNA grapevine virus, grapevine pararetrovirus, grapevine germplasm

## Abstract

In this study, we analyzed the virome of 73 grape samples from two Dagestan ampelographic collections in Russia using high-throughput sequencing of total RNAs. Fourteen viruses and four viroids were identified, with one to eleven of them detected in each plant. For the first time in Russia, we identified grapevine leafroll-associated virus 7 and grapevine Kizil Sapak virus. A total of 206 genomes of viruses and viroids were obtained, and their phylogenetic analysis was carried out. The de novo assembly and tblastx analysis allowed us to obtain contigs of a novel (+) ssRNA genome of a plant virus from the genus *Umbravirus*, which was tentatively named grapevine umbra-like virus (GULV), as well as contigs of a novel dsDNA pararetrovirus from the genus *Caulimovirus*, which was tentatively named grapevine pararetrovirus (GPRV). Complete genomes of these viruses were obtained and used for Sequence Demarcation Tool (SDT) analysis and phylogeny studies. GULV and GPRV were detected in 16 and 33 germplasm samples from the Dagestan collections, respectively.

## 1. Introduction

The grape is one of the oldest crops in the world [1]. The process of its cultivation and domestication took place between the seventh and the fourth millennium BC, and the history of grape consumption goes back over 6000 years [2,3,4,5]. Over such a long period of cultivation, the grapevine can be infected by more than 90 viruses and virus-like agents [6,7], which are diverse not only in the manifestation of symptoms in the plant but also in genomic organization. The predominant grapevine viruses are viruses with positive-sense single-stranded RNA ((+) ssRNA) genomes (74 species); there are also negative-sense single-stranded RNA ((–) ssRNA—9 species), double-stranded RNA (dsRNA—2 species) and DNA viruses (8 species) [7,8,9,10,11,12]. Over the past 13 years, the use of high-throughput sequencing (HTS) methods enabled the detection of more than 35 new grapevine viruses, including 21 species with (+) ssRNA genomes. (+) RNA viruses can be causal agents of economically important diseases, such as infectious degeneration and decline, grapevine leafroll disease, rugose wood and fleck disease [13]. Pathogenic viruses not included in these complexes were described (grapevine Pinot gris virus (GPGV) and grapevine berry inner necrosis virus (GINV)) [13]. It is worth noting that most viruses can cause latent infections. The pathogenicity of a number of viruses (for instance, the recently discovered grapevine Kizil Sapak virus (GKSV), grapevine stunt virus (GSV), grapevine cryptic virus (GCV), etc.) has not yet been established [7,14,15].

Using HTS, four grapevine viruses with circular single-stranded DNA (ssDNA) genomes and three viruses with double-stranded DNA (dsDNA) genomes have been discovered [6,7]. Among them, there are representatives that cause vein clearing and vine decline disease (grapevine vein clearing virus (GVCV) [16,17], Roditis leaf discoloration disease (grapevine Roditis leaf discoloration-associated virus (GRLDaV)) [18] and red blotch disease (grapevine red blotch virus (GRBV)) [19,20].

The cultivation of wine and table varieties for wine production and food purposes makes grapes an important agricultural crop [21,22]. Breeding seedless, high-yielding, disease- and pest-resistant new varieties is one of the important areas of grape selection [23,24]. For breeding needs, genetic resources or germplasms presented in the form of ex situ collections can be used [25]. Such vineyards are a valuable source of potentially useful genes for breeding new grape varieties in selection programs [26,27,28]. In addition, the maintenance of grape varieties of local selection in germplasm collections may contribute to the accumulation of previously unknown pathogens, including viruses.

Ampelographic collections grow all over the world [29,30]. Russia hosts an important portion of the total number of the world’s grapevine genetic resources, which are located in various regions of the country: the Rostov Oblast, the Krasnodar Krai, the Republic of Crimea and the Republic of Dagestan [31].

The Republic of Dagestan is one of the oldest viticultural regions in Russia, and it has a variety of natural landscapes suitable for growing grapes of both wine varieties and collection plantations [32,33]. Currently, two collections of grape germplasm are maintained in the territory of the republic. The Ampelographic Collection of the Dagestan Experimental Station of the N.I. Vavilov Institute of Plant Genetic Resources, which was founded in 1975, maintains about 320 cultivated grape varieties (of which 82 varieties are the products of local selection) and 25 ecotypes of wild forms [34,35]. The second ampelographic collection was founded in 1934 on the basis of the Dagestan Experimental Selection Station of Viticulture and Olericulture; it includes 418 varieties and hybrid forms of grapes [36]. On the basis of these ampelographic collections, research is carried out aimed at preserving and maintaining available collections of grape varieties, improving the grape assortment and certification of existing varieties [35,37,38]. Therefore, information about the presence of viruses in a plant is crucial for obtaining virus-free planting material for further use in selection and nursery breeding as well as establishing new plantations.

Given these issues, the aim of this study was to determine the presence and distribution of viruses and viroids as well as their genetic variability in grape samples from the Dagestan ampelographic collections. Using metagenomic methods, we analyzed the viromes of 73 varieties and hybrids of grapes and identified 14 viruses and 4 viroids. For the first time in Russia, we identified grapevine leafroll-associated virus 7 (GLRaV-7) and GKSV. In addition, we obtained the complete nucleotide sequence of the genome of a novel (+) ssRNA virus tentatively named grapevine umbra-like virus (GULV, genus *Umbravirus*). We also report the discovery of the complete genome of a novel dsDNA virus in grapes that was tentatively named grapevine pararetrovirus (GPRV, genus *Caulimovirus*).

## 2. Materials and Methods

### 2.1. Plant Material and Preparation of mRNA Libraries

In August 2018, 73 grapevine samples with symptoms of viral diseases were collected at the Ampelographic Collection of the Dagestan Experimental Station of the N.I. Vavilov Institute of Plant Genetic Resources (46 samples) and the Dagestan Experimental Selection Station of Viticulture and Olericulture (27 samples) (Supplementary Table S1).

Total RNA was isolated from 1 g samples of shoots and leaves using the CTAB-LiCl method [39]. The isolated RNA was treated with DNase I (Thermo Fisher Scientific, Waltham, MA, USA) and RiboMinus Plant Kit (Thermo Fisher Scientific, Waltham, MA, USA) according to the manufacturer’s instructions. RNA concentration was measured on a Qubit 3.0 fluorometer (Invitrogen, Waltham, MA, USA) using the Qubit RNA BR Assay Kit (Thermo Fisher Scientific, Waltham, MA, USA). Libraries were prepared using the QIAseq Stranded RNA Library Kit (Qiagen, Hilden, Germany). The quality of the prepared libraries was verified on a Qsep1 capillary electrophoresis system (BiOptic, New Taipei, Taiwan), and their concentration was measured on a Qubit 3.0 fluorometer using the Qubit^®^ DNA HS Assay Kit (Thermo Fisher Scientific, Waltham, MA, USA). The 73 prepared libraries were sequenced on a NovaSeq 6000 platform (Illumina, San Diego, CA, USA), producing 150 bp paired-end reads. FASTQ raw sequencing data were deposited to the Sequence Read Archive (SRA) (accession number: PRJNA899472).

### 2.2. Analysis of HTS Data and Assembly of Viral Genomes

The RNA-seq raw data were processed using the Geneious Prime v. 2022.0.1 software package (Biomatters, Auckland, New Zealand). Processing of raw reads consisted of removing adapters and low-quality reads using the BBDuk Trimmer tool as well as deduplication of paired reads and their merging. The processed reads were mapped using the Geneious mapper option with medium-low sensitivity and default parameters to the reference genomes of grapevine viruses and viroids (available on 20 June 2022). In parallel, de novo assembly was carried out using SPAdes and Geneious assemblers. The resulting contigs were subjected to tblastx analysis against the NCBI database of reference viral genomes (upload date: 21 July 2021). For plant viruses, contigs were counted with E-value 1 × 10^–40^ cut off, and for viroids, contigs were counted with E-value 1 × 10^–10^ cut off. Each detected plant virus contig was subjected to blastn analysis against the NCBI database. Samples were considered positive for viruses if more than 10 reads (50 reads for grapevine fanleaf virus (GFLV)) presented. Complete virus genomes were assembled by mapping processed reads to the genomes of reference or closest isolates. The sequences were deposited in GenBank under accession numbers listed in Supplementary Table S2. The percentage of identity of the complete genomes of the detected viruses and viroids with the closest organisms from the GenBank was determined using the blastn tool (Supplementary Table S2).

### 2.3. Validation of Grapevine Viruses and Viroids

To validate mRNA-Seq predicted viral pathogens, we performed reverse transcription PCR (RT-PCR) and quantitative RT-PCR (RT-qPCR). Isolated RNA was used as a template for RT-PCR with random hexamers and RevertAid H Minus Reverse Transcriptase (Thermo Fisher Scientific, Waltham, MA, USA) according to the manufacturer’s protocol. A region of the 18S rRNA gene was chosen as an endogenous control of cDNA synthesis. For RT-PCR, the reaction mixture contained 1× Taq Buffer with (NH_4_)_2_SO_4_, 2.5 mM MgCl_2_, 0.2 mM of each dNTP, 1 µM of each primer, and 0.375 U of Taq polymerase (Thermo Fisher Scientific, Waltham, MA, USA). Forward and reverse sequencing primers for viruses and viroids are listed in Supplementary Table S3. Amplification products were visualized in 1.2% agarose gel. Amplicon sizes were determined by comparison with 100+ bp DNA Ladder (Evrogen, Moscow, Russia). The PCR products of each virus and viroid were validated by bidirectional Sanger sequencing using the BigDyeTM Terminator v3.1 Cycle Sequencing Kit (Thermo Fisher Scientific, Waltham, MA, USA) on an ABI PRISM 3730 automated sequencer (Applied Biosystems, Foster, CA, USA) according to the manufacturer’s instructions. The quality of sequencing data was assessed using the Finch TV 1.4.0 software Geospiza Inc., Seattle, WA, USA) [40] and MEGA 11 software [41]. The assembled nucleotide sequences after verification by the blastn analysis were deposited to the GenBank (Supplementary Table S4).

For detection of GFLV, hop stunt viroid (HSVd) and grapevine yellow speckle viroid 1 (GYSVd-1), TaqMan^®^ RT-qPCR was performed. The reaction mixture contained 1x BioMaster HS-qPCR mix (Biolabmix, Novosibirsk, Russia), 150 nM of each primer and 200 nM of probe. The sequences of primers and probes are listed in Supplementary Table S5. To determine the main PCR parameters in the simplex reaction (efficiency, slope, R^2^ and Y-intercept), the previously described method was used [42]. Three technical replicates were made for each sample. Amplification conditions were as follows: 95 °C for 5 min followed by 50 cycles of 95 °C for 15 s and 60 °C for 60 s. Analysis of RT-qPCR results was performed using the LightCycler 96 SW1.1 software (Roche, Mannheim, Germany).

### 2.4. Assembly of New Virus Sequences and Analysis of their Genomes

To determine the 5′ and 3′ untranslated regions of the GULV genome, the rapid amplification of cDNA ends (RACE) using the Mint RACE cDNA amplification set (Evrogen, Moscow, Russia) was performed. Synthesis of double-stranded cDNA sequences with the addition of a PlugOligo adapter and oligo(dT) 3′ primer was performed for total RNA according to the manufacturer’s instructions using gene-specific primers designed for the known GULV sequence (Supplementary Table S3). To cover the 5′ end, two rounds of amplification were performed, and for the 3′ end, three rounds were performed according to the manufacturer’s protocol. The PCR products were gel-purified and ligated into the pAL2-T plasmid vector using the Quick-TA kit according to the manufacturer’s instructions (Evrogen, Moscow, Russia). For 3–4 transformants per sample, bidirectional Sanger sequencing of the inserts was performed. The evaluation of sequencing data and the assembly of the nucleotide sequences of the GULV isolates were performed using the Finch TV and MEGA 11 software. To determine the similarity between the assembled sequences and sequences available from the GenBank, the blastn analysis was used. The GULV isolates were deposited in GenBank (Supplementary Table S2).

To obtain the full-length viral genome of the novel dsDNA virus GPRV, 22 primer pairs were selected, the PCR products of which completely overlap the bioinformatically predicted consensus sequence (Supplementary Table S3). For PCR with these primers, we used a DNA template present in the total RNA used to prepare the libraries. The PCR mixture contained the same components as described in Section 2.3. PCR fragments of the expected size were obtained and sequenced by the Sanger method using two primers. The GPRV nucleotide sequence was assembled using the Finch TV and MEGA 11 software, and its similarity to the sequences available in the GenBank was determined using the blastn analysis. The nucleotide sequence of the GPRV genome was deposited in GenBank (Supplementary Table S2).

Pairwise comparisons of the complete nucleotide and amino acid sequences of GULV and GPRV with the closest members of the genera *Umbravirus*, *Caulimovirus* and *Ruflodivirus* available at the International Committee on Viral Taxonomy [43] and the NCBI Taxonomy [44] databases were carried out using the Clustal W alignment algorithm in the Sequence Demarcation Tool (SDT v1.2) software [45,46] (Supplementary Tables S6 and S7). To annotate the genomes of GULV and GPRV viruses, we performed a search for coding regions using the on-line available OFRfinder tool (accessed on 23 October 2022) [47] and determination of protein functions using the InterPro tool (accessed on 23 October 2022) [48].

### 2.5. Phylogenetic Analysis and Genetic Diversity

Phylogenetic relationships were determined for the world isolates available as of 25 August 2022 in the GenBank and Russian isolates assembled at more than 90% (Supplementary Table S8). For multiple sequence alignment, the ClustalW method was used. Neighbor-joining (NJ) phylogenetic trees [49] were created by the Maximum Composite Likelihood method using the MEGA 11 software. The statistical significance of branching was assessed with 1000 bootstrap replicates. Clustering of Russian isolates with representative isolates (Supplementary Table S9) on the dendrogram was a criterion for determining molecular groups of nucleotide sequences of envelope proteins and/or complete virus genomes.

## 3. Results

### 3.1. mRNA-Seq Data Analysis

As a result of HTS of 73 libraries, more than 907 million raw reads were generated, with an average of 11 million reads per library; after preprocessing, their number decreased to 2.4 million (Supplementary Table S10). After de novo assembly by SPAdes, an average of 5476 contigs (N50 from 513 to 4641) was obtained, while after de novo assembly by Geneious, an average of 96,695 contigs (N50 from 340 to 456 bp) was obtained (Supplementary Table S10).

Based on the tblastx analysis of contigs assembled by SPAdes, we identified 12 viruses and 2 viroids, compared with 14 viruses and 4 viroids in contigs assembled by Geneious (Supplementary Table S11). For viroid identification, we lowered the threshold E-value from 1 × 10^–40^ to 1 × 10^–10^. This allowed us to identify contigs of Australian grapevine viroid (AGVd), grapevine yellow speckle viroid 2 (GYSVd-2), GYSVd-1 and HSVd.

As a result of de novo assembly of reads and/or mapping of reads to reference virus genomes, we obtained 206 complete or nearly complete genomes for eleven viruses and four viroids (Supplementary Table S2).

### 3.2. Identification of Known Grapevine Viruses and Viroids in the Germplasm Virome

#### 3.2.1. Family: Secoviridae

##### Grapevine Fanleaf Virus

GFLV is a member of the genus *Nepovirus* [6]. It is associated with fanleaf degeneration disease and is one of its most economically important viruses [50,51]. In Russia, GFLV has been found in the Krasnodar Krai and the Republic of Crimea [52,53,54].

The GFLV genome is represented by a bipartite (+) ssRNA [55]. As a result of de novo assembly, we identified GFLV contigs for RNA1 and RNA2 in 38 samples and contigs for two other representatives of the *Nepovirus* genus, arabis mosaic virus (ArMV) and grapevine deformation virus (GDefV), in 11 and 41 samples, respectively. Mapping of preprocessed library reads to reference genomes also revealed the presence of these three viruses. As a result of additional verification, we showed that the ArMV and GDefV reads mapped to the GFLV genome. In addition, the blastn analysis of the detected ArMV and GDefV contigs showed their similarity to GFLV, which allowed us to confirm the presence of only GFLV in the studied samples. The obtained result is explained by the phylogenetic relationship of these viruses belonging to subgroup A of the *Nepovirus* genus and the similarity of their genomes [13,55,56,57,58,59]. Thus, as a result of bioinformatics processing, GFLV was identified in 38 samples. As a result of validation of the samples by RT-PCR and RT-qPCR, its presence was confirmed in 51 libraries (Supplementary Table S11).

When mapping the reads from each library to the reference GFLV genome, we found that the indicator of identical sites was at the level of 48–56%, which is lower than for most other viruses (81% on average). This may be due to the presence of multiple GFLV genetic variants in a single sample. In such cases, after de novo assembly by the SPAdes assembler, there were several contigs corresponding to RNA1 and RNA2 which were used for further phylogenetic analysis. The percentage of similarity of Russian isolates at the nucleotide level with the world’s isolates from the GenBank was 81.00–90.18% for RNA1 and was 84.83–93.10% for RNA2 (Supplementary Table S2).

A dendrogram with RNA1 sequences showed that Dagestan isolates, with bootstrap support of 99, formed a clade distinct from the world’s isolates out of different geographic regions (Supplementary Figure S1a,b). An exception was the isolate D1435e_4 which, with bootstrap support of 89, clustered with the French isolate MH383240.1. Additionally, three isolates (D1420_7, D1420_14 and D1420_15) belong to another clade along with isolates from Switzerland, USA and France.

On the dendrogram with RNA2 nucleotide sequences, Russian isolates formed a distinct cluster with bootstrap support of 72, which also included isolates from Iran, China and Japan (Supplementary Figure S2a,b). An exception was the Dagestan isolate D1421f, which formed a clade with isolates from Italy, France, Turkey and Switzerland. On the dendrogram, there is also a clade of Dagestan isolates D1435f_58 and D1435f_62, which is distinct from the world’s isolates and which also includes French isolates.

In 10 libraries where GFLV was detected, we found satellite RNAs (satRNAs), which, according to the results of tblastx, had a similarity at the nucleotide level with GFLV satRNAs in four libraries (Q37, Q46, Q74 and Q78), had similarity with ArMV satRNAs in four other libraries (Q32, Q34, Q39 and Q42) and had similarity with both GFLV satRNAs and ArMV satRNAs in two other libraries (Q36 and Q38). However, ArMV satRNA reads mapped to GFLV satRNA, which allowed us to confirm the presence of only GFLV satRNAs in these samples. Thus, using a bioinformatics approach, GFLV satRNAs were detected in 10 libraries.

As a result of mapping the reads of each library, the coverage of the reference GFLV satRNA for some sequences ranged from 8.4% to 64.1% and was more than 93% for the rest. Mapping the reads to the nucleotide sequence of satRNA of the nearest isolate made it possible to increase the percentage of assembly for all isolates to 94.5–99.0%.

The blastn analysis showed the similarity of Russian GFLV satRNA sequences both with the world’s GFLV satRNA isolates and with ArMV satRNA isolates. For the GFLV isolates identified in the libraries Q36, Q37, Q74 and Q78, satellite RNAs had similarity both with ArMV satRNA sequences (90.85–95.25%) and with GFLV satRNA sequences (90.14–94.10%). On the dendrogram, these Russian sequences are grouped close to each other with bootstrap support of 87, and they form a distinct clade with satellite RNAs of ArMV and GFLV isolates (Supplementary Figure S3). For satRNAs of another group of isolates (Q32, Q34, Q38, Q39, Q42 and Q46), as shown by the blastn analysis, the closest sequences were GFLV satRNAs (pairwise identity of 90.44–94.01%). The identity with ArMV satRNA was 86.35–87.56%. On the dendrogram, these sequences, with bootstrap support of 100, were grouped with the GFLV satRNA isolate KC900164.1 from South Africa, and with bootstrap support of 75, they formed a clade with ArMV satRNA sequences. For the GFLV satRNA isolate Q34, the closest sequence was the sequence of the French isolate KX034958.1. On the dendrogram, it also clusters separately from other Russian isolates.

Based on the results of validation, 13 samples were found to be positive for GFLV satRNA, which was confirmed by Sanger sequencing (Supplementary Table S8). SatRNA was identified in 34% of GFLV isolates, which supports its presence in only a fraction of the world’s isolates [13]. In this study, we discovered for the first time in Russia GFLV isolates containing satellite RNAs.

#### 3.2.2. Family: Betaflexiviridae

The Betaflexiviridae family currently includes four genera (*Fivirus*, *Foveavirus*, *Trichovirus* and *Vitivirus*), the representatives of which infect grapes worldwide [7]. Grapevine rupestris stem pitting-associated virus (GRSPaV), grapevine virus T (GVT) (genus *Foveavirus*), GPGV (genus *Trichovirus*) and grapevine virus A (GVA,genus *Vitivirus*) have been detected in most vineyards in Russia [30,53,54,60]. In 2019, a novel member of the proposed genus “*Fivivirus*”, GKSV, was identified on an asymptomatic grapevine cv. Kizil Sapak in the USA collection [14].

##### Grapevine Rupestris Stem Pitting-Associated Virus

Based on bioinformatics analysis, GRSPaV was identified in 65 samples (Supplementary Table S11). When mapping the reads of each library to the reference GRSPaV genome (NC001948.1), the coverage did not exceed 90% for a number of isolates. Read mapping to the genome of the closest isolate allowed us to obtain 48 complete GRSPaV genomes (Supplementary Table S12). The blastn analysis showed that the percentage of identity of the Russian isolates with the world’s isolates was 88.80–98.72%. During validation with a pair of primers targeting the coat protein (CP) region of the genome (Supplementary Table S3), GRSPaV was detected in 65 samples, which confirms the results of bioinformatics processing.

Phylogenetic analysis was performed for 163 complete genomes of the world’s isolates from NCBI and 48 from this study (Supplementary Figure S4a–c). On the dendrogram, Dagestan isolates with high bootstrap support clustered with representative isolates belonging to GRSPaV molecular groups I, II and III according to previously defined molecular classification [61]. Molecular group I included 21 Dagestan isolates which corresponded to sub-groups T, U and V (Supplementary Figure S4c). Group II included 24 Dagestan isolates divided into sub-clades 2a, 2b and 2c (Supplementary Figure S4b). The isolates that we found were also part of sub-groups D and E. Moreover, six isolates (D1431k, D1433k, D1434k, D1421k, D1425k and D1371k) formed a subgroup distinct from the rest of the world’s isolates. In addition, the isolate D1283k was grouped with the isolate A1557k found in the Anapa collection. Molecular group III included three Dagestan isolates, two of which (D1290k and D1387k) clustered with representative isolates from sub-group M, while isolate D1386k belonged to sub-group L (Supplementary Figure S4a).

##### Grapevine Pinot Gris Virus

Based on bioinformatics analysis, GPGV was identified in 39 samples. GPGV validation was performed with two primer pairs (Supplementary Table S3). Using the primer pair GPgCP_6742F/GPgCP_7143R, we confirmed the presence of GPGV in 36 samples as well as in four additional libraries (Q54, Q61, Q65 and Q73) (Supplementary Table S11). Another five samples (Q42, Q46, Q55, Q91 and Q94) were shown to be positive using the second pair of primers GPG-6609F/GPG-7020R.

We assembled 27 complete genomes of the virus; their identity with the nearest isolates from the GenBank was 97.91–99.03% (Supplementary Table S2). Based on phylogenetic analysis, it was shown that the Russian isolates belong to the same cluster and are grouped with isolates from Russia, Germany, Italy, Slovakia and USA (Supplementary Figure S5). An exception is isolates D1446h, D1434h and D1425h which belong to a subclade distinct from other Russian isolates.

We carried out an analysis of the 3′ region of the movement protein (MP) gene which may play a role in the manifestation of symptoms of GPGV infection [62]. It was previously found that the MP gene may have C to T substitutions at position 6685 and at position 6688 [63,64]. In our study, all Russian isolates contained C at position 6685, and, therefore, MP was six amino acids longer than in the reference sequence (NC_015782.2). One isolate (Q31) contained T at position 6688, resulting in a five amino acid shorter MP than in the reference sequence (Supplementary Figure S6a,b). Some of the plants we studied had GPGV symptoms (Supplementary Table S1). However, they were all simultaneously affected by several viruses and/or viroids. This fact does not allow us to establish a clear correlation between symptoms, the presence of GPGV in the plant and polymorphism of the 3′ end of MP.

##### Grapevine Virus A

In our study, GVA was bioinformatically detected and validated in two samples (Supplementary Table S11). To assemble the complete virus genome sequence, we mapped the reads of each library to the genome of the nearest isolate, which allowed us to obtain two complete genomes (Supplementary Table S12). The blastn analysis showed that the Russian isolate D1371o had a 79.83% identity at the nucleotide level with an isolate from South Africa (MW309530.1), while the isolate D1357o had an 87.92% identity with a French isolate (MG925333.1). In previous studies, we have also shown a rather low (at the level of 81.41–85.97%) identity of Russian isolates with the world’s isolates [30]. On the dendrogram, the Russian GVA isolates were grouped with representative isolates of molecular group I (Supplementary Figure S7), clustering with isolates from France, South Africa and Russia.

##### Grapevine Virus T

GVT was detected bioinformatically in five libraries (Supplementary Table S11; contigs of Panax ginseng flexivirus 1 correspond to GVT due to the lack of GVT in the refseq database). We confirmed the presence of GVT in six libraries using RT-PCR. For three isolates (D1423s, D1385s and D1357s), we obtained the complete virus genomes (Supplementary Table S11). Pairwise identity at the nucleotide level with an isolate from Japan (LC617948.1) ranged from 96.51 to 97.41% (Supplementary Table S2). On the dendrogram, the Russian isolates clustered with the Japanese isolate and formed a new GVT molecular group VIII (Supplementary Figure S8).

##### Grapevine Kizil Sapak Virus

Based on the tblastx analysis, contigs of five representatives of the *Trichovirus* genus were identified in two libraries (Q65 and Q84): grapevine berry inner necrosis virus (GINV), apple chlorotic leaf spot virus (ACLSV), peach mosaic virus (PMV), cherry mottle leaf virus (CMLV) and apricot pseudo-chlorotic leaf spot virus (APCLSV) (Supplementary Table S11). The blastn analysis showed the similarity of these contigs to the only GKSV species which was not detected by tblastx due to the lack of a nucleotide sequence in the refseq database. When mapping the reads of each library to the genome of the GKSV isolate (LC541734), we found it not only in the Q65 and Q84 libraries (over 80% coverage of the reference genome with reads), but also in the Q64 library (21.4% coverage). RT-PCR with a pair of primers designed for the CP region of the genome confirmed the results of the bioinformatics analysis.

Mapping the reads to the genome of the nearest isolate made it possible to increase the percentage of genome assembly for two GKSV isolates: D1365u and D1297u (Supplementary Table S12). Pairwise identity at the nucleotide level with the closest isolate from France (MK204653.1) was 87.15% and 87.65%, respectively (Supplementary Table S2). On the dendrogram, the Russian isolates clustered near each other with bootstrap support of 99% and constituted a distinct clade together with the French (MK204653.1) and Turkmenistan (MN172166.1) isolates (Supplementary Figure S9).

#### 3.2.3. Family: Tymoviridae

The Tymoviridae family includes representatives of three genera that infect grapes: *Marafivirus*, *Maculavirus* and *Gratylivirus* [7]. The most widespread viruses are grapevine rupestris vein feathering virus (GRVFV), grapevine asteroid mosaic-associated virus (GAMaV), grapevine Syrah virus 1 (GSyV-1) (genus *Marafivirus*), grapevine fleck virus (GFkV) and grapevine red globe virus (GRGV) (genus *Maculavirus*). At present, all these viruses have been detected in Russian vineyards, including the Anapa ampelographic collection [30,53,54].

##### Grapevine Fleck Virus

Based on bioinformatics analysis, GFkV was identified in two libraries (Q39 and Q53). RT-PCR confirmed the presence of target amplicons in the two libraries as well as in six additional ones (Q25, Q33, Q50, Q51, Q57 and Q71) (Supplementary Table S11). Complete genomes for the detected GFkV isolates could not be obtained.

##### Grapevine Rupestris Vein Feathering Virus

Based on bioinformatics analysis, GRVFV contigs were identified in 13 libraries (Supplementary Table S11). Complete genomes for the identified GRVFV isolates could not be obtained. Validation of the samples was carried out using two pairs of primers (Supplementary Table S3). Using the primer pair GRVFV_F/GRVFV_6598R, we confirmed the presence of GRVFV in 13 libraries as well as in eight additional samples (Q61, Q63, Q65, Q66, Q68, Q70, Q75 and Q89). The use of the other primer pair GRVFV6155-F/GRVFV6598-R allowed us to obtain the target product for Q67 and Q80 samples. Thus, GRVFV was identified in 23 samples.

##### Grapevine Red Globe Virus

Based on the tblastx analysis, we identified GRGV contig in one sample (Q73). As a result of RT-PCR, fragments of the expected size were obtained for this sample as well as for six additional ones (Q24, Q25, Q47, Q59, Q65 and Q89).

#### 3.2.4. Family: Closteroviridae

Currently, three genera in the Closteroviridae family are distinguished that infect grapevines worldwide [6,65]. Typical representatives of the genera *Ampelovirus* (grapevine leafroll-associated virus 1, 3, 4 and 13 (GLRaV-1, GLRaV-3, GLRaV-4 and GLRaV-13)) and *Closterovirus* (grapevine leafroll-associated virus 2 (GLRaV-2)) cause economically important grapevine leafroll disease (GLRD), while grapevine leafroll-associated virus 7 (GLRaV-7) infection (genus *Velarivirus*) does not manifest or displays vague symptoms of leafroll on the plant [6,66]. Co-infection of GLRaV-7 with other closteroviruses prevents GLRaV-7 from being identified as the etiological agent of GLRD [67]. GLRaV-1, GLRaV-2, GLRaV-3 and GLRaV-4 have previously been detected in Russia [30,53,54,68,69].

##### Grapevine Leafroll-Associated Virus 1

Based on bioinformatics analysis, GLRaV-1 was identified and then validated in two libraries (Q59 and Q73). When mapping the reads of each library to the reference sequence, we obtained the complete genome for the isolate D1371d (94.2% coverage). For an isolate from the Q73 library, we obtained a partial genome sequence with a coverage of 83.1%. Mapping the reads to the genome of the isolate closest to the one we identified made it possible to increase the completeness of the genome assembly to 86.9% (Supplementary Table S12). The blastn analysis showed a 90.08% identity of the isolate D1371d with the nearest isolate from the GenBank (MH807218.1). On the dendrogram obtained from the analysis of 28 complete genome sequences of GLRaV-1 isolates, the isolate D1371d formed a clade with other isolates from Russia and USA (Supplementary Figure S10). Determination of GLRaV-1 molecular groups was performed on a sample of 227 nucleotide sequences of the coat protein gene (Supplementary Figure S11). Based on phylogenetic analysis, the Dagestan isolate D1371d was assigned to group II.

##### Grapevine Leafroll-Associated Virus 7

Based on bioinformatics analysis, GLRaV-7 was identified in four libraries (Q64, Q65, Q68 and Q84) (Supplementary Table S9) and detected using a pair of primers designed for the genomes we identified. Sanger sequencing confirmed these results (Supplementary Table S8). Thus, for the first time in Russia, we identified GLRaV-7 in four grape samples.

Complete genomes of GLRaV-7 were obtained in all four libraries. The blastn analysis showed that pairwise identity at the nucleotide level with isolates from the USA (JN383343.1) and Albania (HE588185.1) comprises 90.53–96.72%. Based on phylogenetic analysis, the closest to the Russian isolates D1362i, D1365i and D1297i were found to be Japanese and Pakistani isolates (Supplementary Figure S12). The isolate D1366i was grouped with a USA isolate with bootstrap support of 100.

#### 3.2.5. Family: Unassigned

##### Grapevine Satellite Virus

Grapevine satellite virus (GV-Sat) is a putative member of the *Virtovirus* genus that infects grapevines [7]. In Russia, this pathogen has been detected in a collection in the Krasnodar Krai [30]. In our study, we identified the nucleotide sequences of GV-Sat and validated its presence in the Q59 library (Supplementary Table S11). We assembled the complete genome of the isolate D1371m, for which the level of identity with a USA isolate (NC_021480.1) comprised 96.07%. On the dendrogram, the identified GV-Sat isolate clustered together with this same reference isolate (Supplementary Figure S13). In addition to GV-Sat, this sample (from the Q59 library) of the Armenian variety Zovuni is co-infected with other pathogens, particularly GVA and GLRaV-1, which may confirm their possible helper function for GV-Sat (Supplementary Table S13) [30,70,71,72]. Because other viruses were also found in our samples, further studies are required to determine their role as helpers for GV-Sat.

#### 3.2.6. Family: Pospiviroidae

Currently, the viroids GYSVd-1, GYSVd-2 and AGVd are assigned to the genus *Apscaviroid*, while HSVd is assigned to the genus *Hostuviroid* [73]. HSVd and GYSVd-1 are widely distributed in vineyards worldwide [74,75], whereas the distribution of AGVd and GYSVd-2 is limited [76,77]. In Russia, all four viroids have been detected in vineyards and collection plantations in the Krasnodar Krai [30,54].

##### Hop Stunt Viroid

Based on the tblastx analysis followed by analysis of contigs with an E-value 1 × 10^–20^ cut off, we identified HSVd contigs in 48 libraries. Changing the E-value threshold to 1 × 10^–10^ made it possible to identify the contig for the Q27 library. HSVd reads were identified in two more libraries (Q50 and Q84). Validation by RT-qPCR allowed us to identify HSVd in 52 samples. The main parameters of RT-qPCR are listed in Supplementary Table S9.

Complete HSVd genomes were obtained for 48 isolates. The percentage of identity of the Russian isolates at the nucleotide level with the nearest isolates from the GenBank was 94.97–100.00% (Supplementary Table S2). Phylogenetic analysis was performed for all complete genomes of HSVd isolates from different cultures available from the GenBank. Based on the analysis, 43 Dagestan isolates were shown to cluster with isolates from the Hop group and seven clustered with isolates from the Plum-Hop/cit3 group (Supplementary Figure S14a,b). In most cases, the host of representative isolates clustering with the Russian isolates was grapevine.

##### Grapevine Yellow Speckle Viroid 1

In our study, GYSVd-1 contigs and reads were identified in 33 libraries. Validation of the obtained results by RT-qPCR made it possible to identify GYSVd-1 in 35 samples. The main parameters of RT-qPCR are listed in Supplementary Table S9.

Complete GYSVd-1 genomes were obtained for 24 isolates. The blastn analysis showed their high identity with the world’s isolates at the level of 97.00–100% (Supplementary Table S2). The most divergent Dagestan isolate Q63 detected on the Uzbek variety Charas muskat was 91.89% identical at the nucleotide level to the isolate KX966273.1. On the dendrogram, the Russian isolates were evenly distributed (Supplementary Figure S15a–d), which confirms their genetic diversity and the results of our previous study [30]. The most similar to the Russian isolates were the isolates from India, Iran, Pakistan, Thailand, Brazil, Croatia, Russia, Canada, Turkey, Australia and Czech Republic. The isolates D1300b and D1289b were grouped with Russian and Slovak isolates, while the group with D1362b, D1305b and D1304b mainly included isolates from Nigeria. Four isolates D1451b, D1427b, D1359b and D1292b, with bootstrap support of 85, formed a distinct clade. The most divergent was the isolate D1296b, which clustered separately from other Dagestan isolates and mainly with Chinese isolates.

##### Grapevine Yellow Speckle Viroid 2

In this study, GYSVd-2 contigs were identified bioinformatically and validated by RT-PCR in four libraries (Supplementary Table S11). We obtained three complete genomes of GYSVd-2 isolates (D1367r, D1365r and D1357r), which were identical at the nucleotide level with other isolates from Russia (MZ803184.1), South Korea (OL634848.1) and Greece (LR735994.1) at the level of 99.44%, 99.72% and 100.00%, respectively. On the dendrogram, the isolates D1367r and D1365r clustered close to isolates from China, Iran and Russia (Supplementary Figure S16a,b). The isolate D1357r formed a group with the Pakistani and Italian isolates.

##### Australian Grapevine Viroid

Based on the tblastx analysis, we identified AGVd contigs and reads in three libraries, which was confirmed by RT-PCR and target amplicon sequencing (Supplementary Table S8). For isolate D1370a, the complete genome was obtained. Its identity at the nucleotide level with an Iranian isolate (KF876038.1) was 98.92%. On the dendrogram, the Russian isolate D1370a clustered with isolates from China and Iran (Supplementary Figure S17).

### 3.3. Discovery of Novel Grapevine Viruses

#### 3.3.1. Family: Tombusviridae

##### Grapevine Umbra-Like Virus

In a previous study on the virome of the Anapa ampelographic collection [30], we identified a 649 bp nucleotide sequence presumably belonging to a novel virus of the genus *Umbravirus*, which was tentatively named grapevine umbra-like virus (GULV).

In this study, as a result of de novo assembly by the SPAdes assembler, we obtained in three libraries (Q28, Q64 and Q82) contigs with a length of 1859 bp, 1048 bp and 3228 bp. The contigs had homology with the nucleotide sequence of the carrot mottle mimic virus (CMoMV), a representative of the *Umbravirus* genus of the Tombusviridae family. The blastn analysis with the megablast option showed similarity with the RdRp GULV (ON669249.1) sequence that we identified earlier [30]. Moreover, the blastn analysis with the “Somewhat similar sequences” option revealed similarities of three contigs with representatives of the *Umbravirus* genus: wheat umbra-like virus (WULV, pairwise identity of 66.42–66.63%, query cover of 35–78%), strawberry virus A (StrVA, pairwise identity of 65.85–67.44%, query cover of 24–77%), papaya virus Q (PpVQ, pairwise identity of 64.90–65.05%, query cover of 24–75%). As a result of mapping the reads of each library to the 3228 bp contig, we identified from 10 to 320 reads in six libraries. The length of the GULV consensus sequence in libraries Q28 and Q64 was increased to 3245 bp and 3178 bp, respectively.

After carrying out RACE, we obtained sequences of 209 bp (isolates Q28 and Q64) and 196 bp (isolate Q82) for the 5′ terminal region of the genome of GULV. Additionally, we obtained sequences of 46 bp for the 3′ terminal region of the isolates Q28 and Q82. Thus, the length of the genomes of GULV isolates Q28, Q64 and Q82 comprised 3343 bp, 3268 bp and 3330 bp, respectively. The complete genome for the Q28 GULV isolate consisted of 3343 bp. The blastn analysis showed their pairwise identity at the nucleotide level with the closest WULV isolate to be at the level of 66.32–66.51% (Supplementary Table S2).

**Figure 1:**
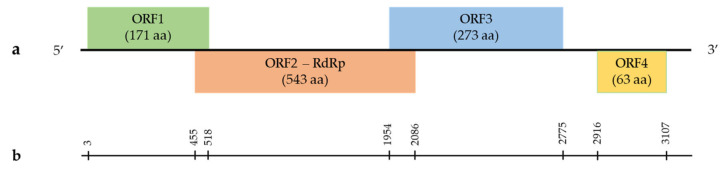
Schematic organization of the grapevine umbra-like virus (GULV) genome. (**a**) The genome contains four predicted ORFs: ORF1 (unknown function), ORF2 (RdRp), ORF3 (unknown function) and ORF4 (unknown function). (**b**) Nucleotide positions of GULV ORFs.

The genome of umbraviruses is represented by a linear (+) single-stranded RNA (ssRNA) that has four open reading frames (ORFs) [78]. Based on the analysis of the nucleotide sequence of GULV isolates in the ORF Finder program, four ORFs were predicted (Figure 1). The 516 bp ORF1 encodes a 171 amino acid (aa) peptide with an unknown function. The blastp analysis showed the highest identity with ORF1 of the WULV sequence (pairwise identity of 45.51–47.02%, query cover of 99–100%). ORF1 overlaps with the 1632 bp ORF2 sequence encoding a 543 aa protein with a predicted function of RNA-dependent RNA polymerase (RdRp). The blastp analysis showed the highest identity at the amino acid level with the RdRp sequences of WULV (pairwise identity of 68.49–68.70%, query cover of 85%) and StrVA (pairwise identity of 59.77–61.19%, query cover of 92–97%). ORF3 (822 bp in length) overlaps with ORF2 and encodes a 273 aa peptide with an unknown function. ORF4 is 192 bp long and encodes a 63 aa peptide.

**Figure 2:**
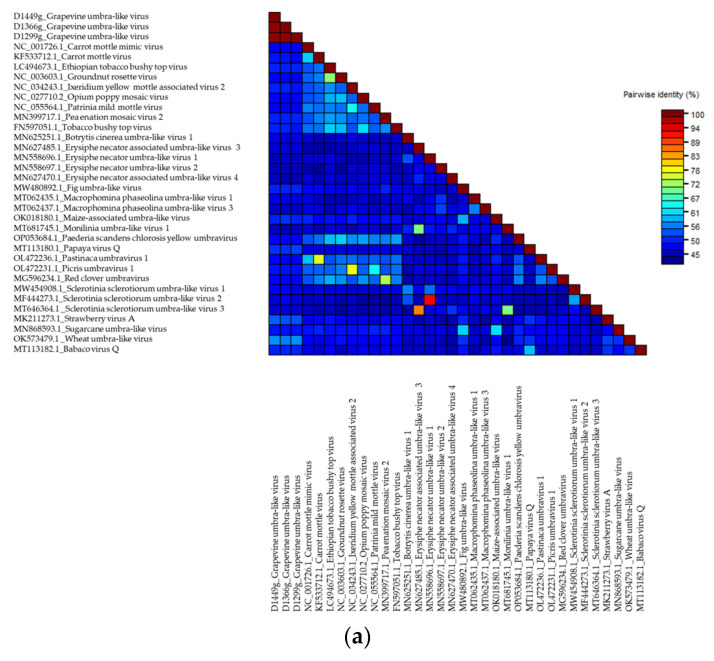

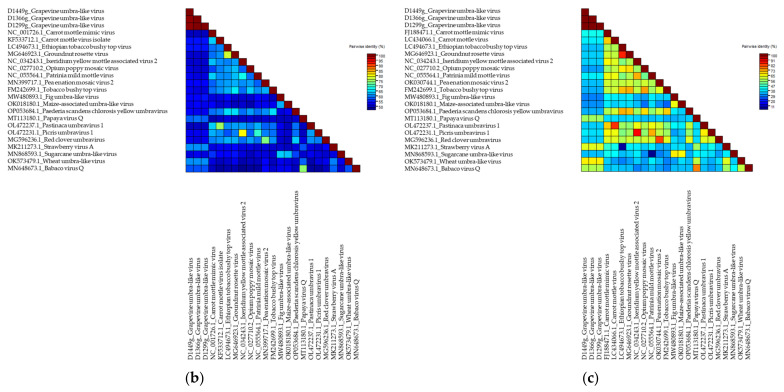
Color-coded pairwise identity matrix including (**a**) complete genomes, (**b**) nucleotide sequences of the RNA-dependent RNA polymerase (RdRp), (**c**) amino acids sequences of the RdRp of grapevine umbra-like virus isolates, representative isolates of the genus *Umbravirus* and unclassified umbra-like viruses. GenBank accession numbers for each isolate are present by virus isolate names and are also in Supplementary Table S6.

**Figure 3:**
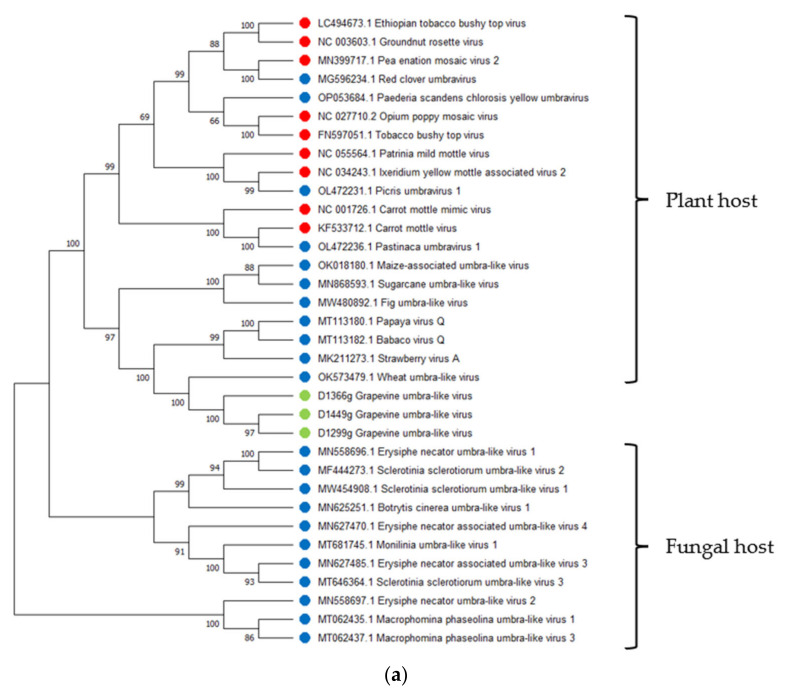

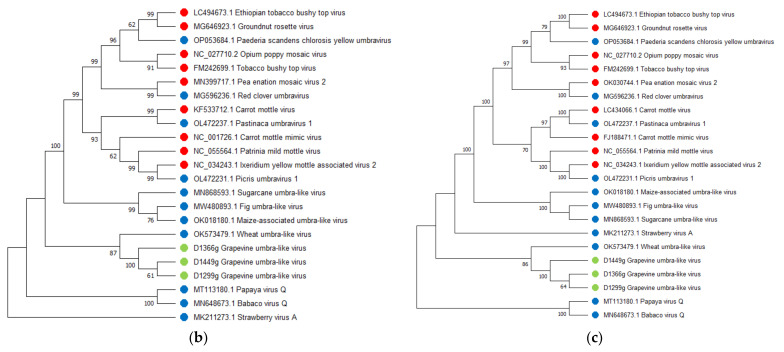
Phylogenetic analysis of (**a**) complete genomes, (**b**) nucleotide sequences of the RNA-dependent RNA polymerase (RdRp) and (**c**) amino acid sequences of the RdRp of grapevine umbra-like virus (marked with a green dot), representatives of the genus *Umbravirus* (marked with a red dot) and unclassified umbra-like viruses (marked with a blue dot). The tree was constructed using the neighbor-joining method. The numbers at the nodes indicate bootstrap values >60% (1000 replicates). GenBank accession numbers for each isolate are present by virus isolate names and are also in Supplementary Table S6.

The SDT analysis was performed for three GULV isolates and 31 complete genomes of representatives of the genus *Umbravirus* and other unclassified umbra-like viruses hosted by plants and fungi (one nearest isolate for each species). The highest identity with GULV on the identity matrix was shown by the genomes of WULV, StrVA and PpVQ, with pairwise identity of 51.5–56.6% (Supplementary Table S14; Figure 2a). The results of the SDT analysis were confirmed by phylogenetic analysis. On the dendrogram (Figure 3a), Russian GULV isolates grouped with each other with bootstrap support of 97–100 and also formed a clade with plant umbra-like viruses WULV, StrVA, PpVQ and babaco virus Q (BabVQ). Viruses hosted by fungi clustered separately.

Pairwise identities of the nucleotide sequences of the RdRp gene (ORF2) and the corresponding amino acid sequences were assessed on a sample of plant viruses. The SDT analysis was performed for the RdRp sequences of three GULV isolates and 20 representatives of umbraviruses and umbra-like viruses. The pairwise identity matrix showed that the closest species to GULV in terms of the nucleotide and amino acid RdRp sequences are WULV, StrVA, PpVQ and BabVQ (Supplementary Tables S15 and S16; Figure 2b,c). The percentage of identity with them varied from 58.4% to 61.8% for nucleotide sequences and from 55.4% to 66.3% for amino acid sequences. On the RdRp dendrograms, GULV clustered as a distinct clade together with WULV with high bootstrap support (Figure 3b,c).

For the detection of GULV in grape samples, we designed primers for two regions of its genome: the sequence of the replicase gene (ORF2) and the ORF3 sequence (Supplementary Table S3). With the first pair of primers GULV_695F/GULV_1268R, we confirmed the presence of the virus in 10 samples. With primers GULV_2191F/GULV_2609R, a product of the expected size was identified in six libraries. The amplicons were sequenced by the Sanger method (Supplementary Table S4).

Plants infected with viruses from the *Umbravirus* genus usually show symptoms of mosaics and mottling [79]. In our study, it was difficult to associate the presence of any specific symptoms with GULV infection due to multiple co-infections of grapevine samples.

Thus, in this study, we identified for the first time the complete genome of an umbra-like virus associated with grapevine. Moreover, the identity of the complete genomes of GULV isolates with the nearest umbraviruses does not exceed 70% [78], which makes it possible to classify GULV as a novel species of the *Umbravirus* genus.

#### 3.3.2. Family: Caulimoviridae

##### Grapevine Pararetrovirus 

Based on de novo assembly by Geneious, we identified in six libraries (Q24, Q50, Q52, Q58, Q73 and Q84) contigs ranging in size from 265 bp to 461 bp that had homology with dsDNA plant pararetroviruses of the Caulimoviridae family: rudbeckia flower distortion virus (RuFDV, genus *Ruflodivirus*) (Q24 and Q58), soybean mild mottle pararetrovirus (SMMP, genus *Caulimovirus*) (Q24) and cauliflower mosaic virus (CaMV, genus *Caulimovirus*) (Q50, Q52). The blastn analysis with the megablast option did not show any similarity with viruses available in the GenBank, which suggested the presence of a new virus in the studied samples. When we used the “Somewhat similar sequences” option, we found similarities with various plant viruses from the genus *Caulimovirus* presented in Table 1.

**Figure 4:**
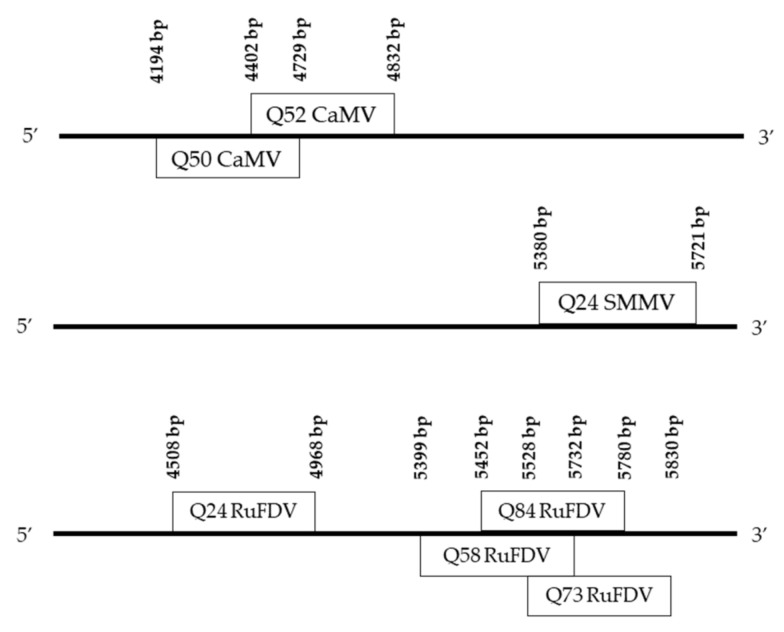
Mapping de novo assembled contigs to reference sequences of cauliflower mosaic virus (CaMV NC_001497.2, genome size of 8024 bp), soybean mild mottle pararetrovirus (SMMV, NC_018505.1, genome size of 7757 bp) and Rudbeckia flower distortion virus (RuFDV, NC_011920.1, genome size of 8222 bp).

The seven identified contigs were mapped to the reference sequences of RuFDV, SMMP and CaMV (Figure 4) and assembled into two contigs corresponding to the 5′ and 3′ ends of the replicase gene. Reads of 73 libraries analyzed in this study, as well as 47 published in the previous study [30], were mapped onto the obtained contigs with a length of 394 bp and 761 bp. Thus, we were able to assemble a 7507 bp consensus sequence. Based on mapping preprocessed reads of each Dagestan germplasm library, positive results were obtained for eight libraries.

The longest nucleotide sequence of 7439 bp was assembled for the Q24 library, with an assembly reference sequence coverage of 86.8% and a pairwise identity of 96.6%. The blastn analysis showed its identity at the nucleotide level with representatives of the genus *Caulimovirus*: plant-associated caulimovirus 1 (PaCV-1) (OL472131.1) (66.90% identical), CaMV (AB863193.1) (65.08% identical) and dahlia mosaic virus (HQ336478.1) (65.08% identical).

Based on Sanger sequencing and assembly of amplicons, we obtained the complete sequence of an 8583 bp dsDNA virus that was tentatively named grapevine pararetrovirus (GPRV). As a result of validation by RT-PCR, the presence of this nucleotide sequence was confirmed in 14 samples in which the reads were identified as well as in 19 additional samples (Supplementary Table S11).

**Figure 5:**
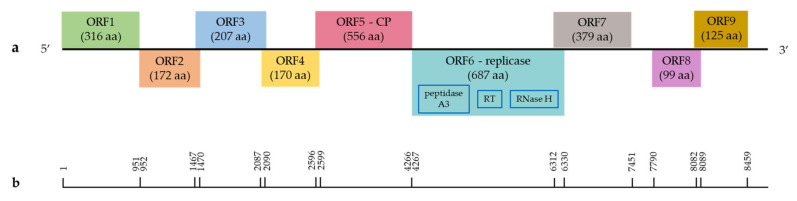
Linear representations of the dsDNA genome of grapevine pararetrovirus (GPRV). (**a**) Predicted open reading frames encoding proteins with unknown function (ORF1-ORF4 and ORF7-ORF9), coat protein (CP, ORF5) and polymerase polyprotein P6 (ORF6) with domains of peptidase A3, reverse transcriptase (RT) and ribonuclease H (RNase H) are shown. (**b**) Nucleotide positions of GPRV ORFs.

The genome of caulimoviruses is represented by non-covalently closed circular double-stranded DNA (dsDNA) comprising at least six ORFs [80,81]. Using the ORF Finder program, nine ORFs were predicted for the GPRV isolate D1454w (Figure 5). For amino acid sequences encoded in ORF1 (nt 1 to 951, putative P1 peptide with a size of 316 aa), ORF2 (nt 952 to 1470, putative P2 peptide with a size of 172 aa), ORF3 (nt 1467 to 2090, putative P3 peptide with a size of 207 aa), ORF4 (nt 2087 to 2599, putative P4 peptide with a size of 170 aa), ORF7 (nt 6312 to 7451, putative P7 peptide with a size of 379 aa), ORF8 (nt 7790 to 8089, putative P8 peptide with a size of 99 aa) and ORF9 (nt 8082 to 8459, putative P9 peptide with a size of 125 aa), protein functions were not predicted by the InterPro program. ORF5 (nt 2596 to 4266) encodes a putative coat protein P5 consisting of 556 aa. ORF6 (nt 4267 to 6330) encodes a putative polymerase polyprotein P6 consisting of 687 aa with peptidase A3, reverse transcriptase (RT) and ribonuclease H (RNase H) domains.

**Figure 6:**
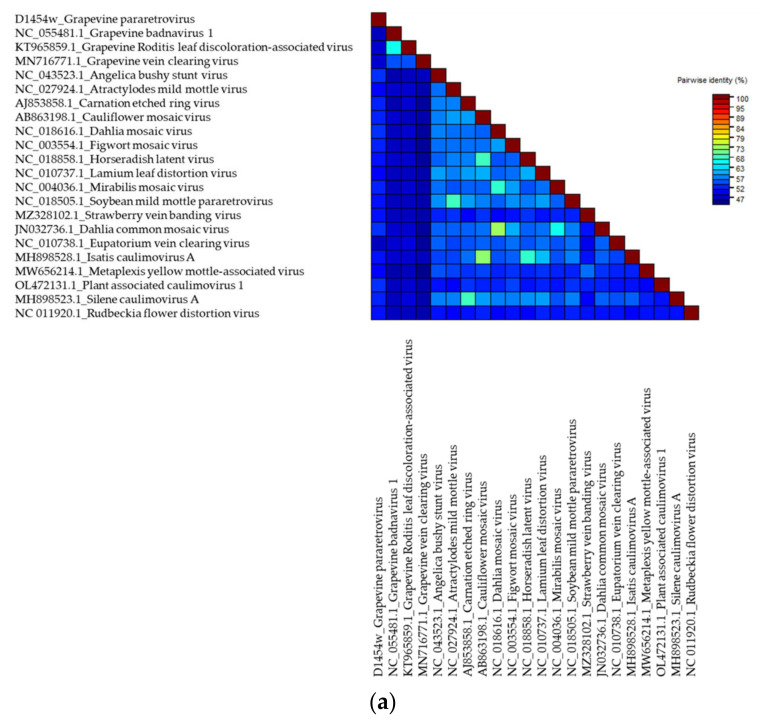

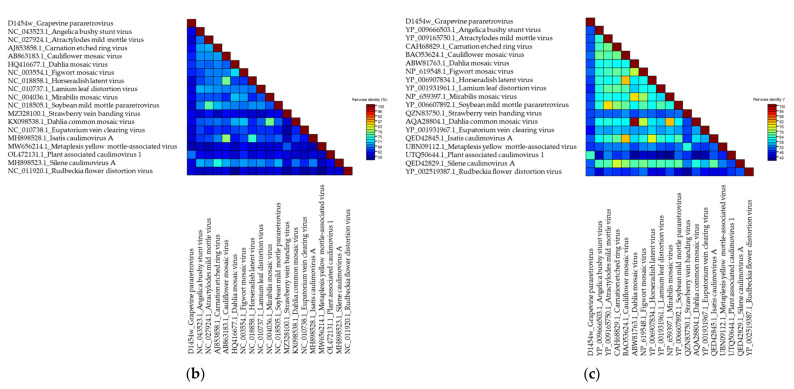
Color-coded pairwise identity matrix including (**a**) complete genomes, (**b**) nucleotide sequences of polymerase region and (**c**) amino acid sequences of polymerase region of grapevine pararetrovirus, representative isolates of the genera *Ruflodivirus*, *Caulimovirus*, including unclassified *Caulimovirus*, and *Badnavirus*. GenBank accession numbers for each isolate are present by virus isolate names and are also in Supplementary Table S7.

**Figure 7:**
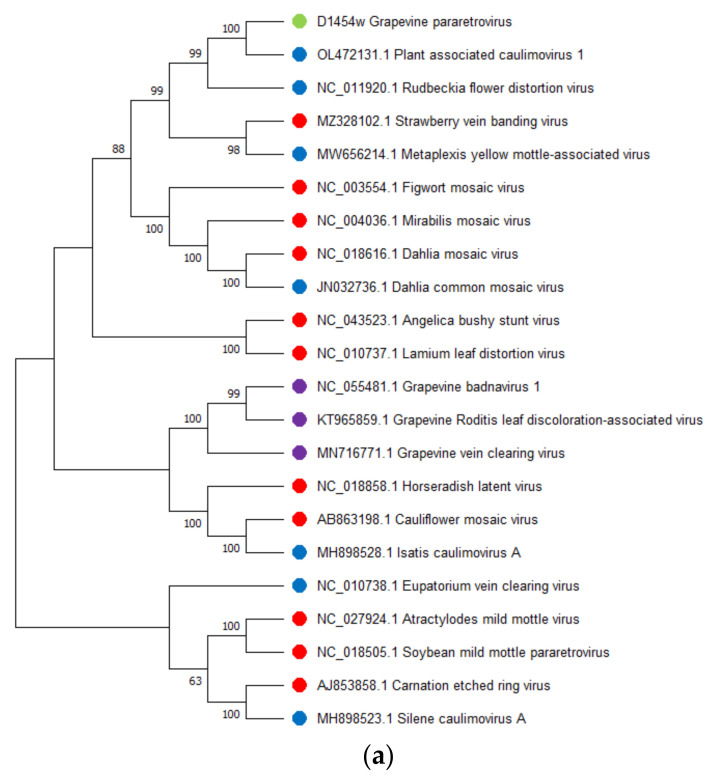

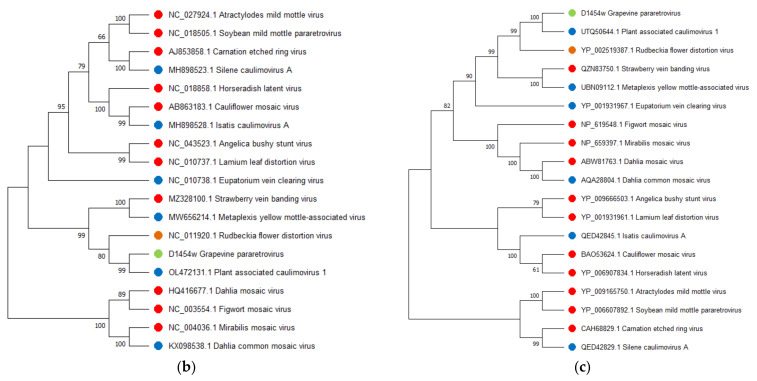
Phylogenetic analysis of (**a**) complete genomes, (**b**) nucleotide sequences of polymerase region and (**c**) amino acids sequences of polymerase region of grapevine pararetrovirus (marked with a green dot), representative isolates of the genera *Ruflodivirus* (marked with an orange dot), *Caulimovirus* (marked with a red dot), including unclassified *Caulimovirus* (marked with a blue dot), and *Badnavirus* (marked with a purple dot). The tree was constructed using the neighbor-joining method. The numbers at the nodes indicate bootstrap values >60% (1000 replicates). GenBank accession numbers for each isolate are present by virus isolate names and are also in Supplementary Table S7.

The SDT analysis was performed for the GPRV sequence and the 21 nearest complete genomes of species from the genera *Ruflodivirus* and *Caulimovirus*, including unclassified caulimoviruses, as well as three species of the genus *Badnavirus* that infect grapes: grapevine badnavirus 1 (GBV-1), grapevine Roditis leaf discoloration-associated virus (GRLDaV) and grapevine vein clearing virus (GVCV). Previously, a 3678 bp contig (MN716782.1) was found in grapevine, which supposedly belonged to a new species of the genus *Caulimovirus* and was named grapevine-associated caulimovirus (GaCV) [82]. The blastx analysis of de novo contigs, as well as the blastn analysis of the resulting GPRV sequence, showed no similarity to GaCV. In addition, the complete genome of this virus is not available at the GenBank, therefore GaCV was not used in SDT and phylogenetic analyses. The SDT analysis showed the highest identity with GPRV at the level of 52.8% for PaCV-1 (Supplementary Table S17, Figure 6a). On the dendrogram, GPRV grouped with PaCV-1 and RuFDV (Figure 7a). Three dsDNA grapevine badnaviruses clustered together but not with the novel GPRV. Therefore, these viruses were not included in further SDT analysis for the polymerase region.

A comparison of the nucleotide and amino acid sequences of the polymerase region was performed for GPRV and 18 viral isolates (representatives of the genera *Ruflodivirus* and *Caulimovirus*). The highest identity with GPRV at the nucleotide level was found for PaCV-1 (65.3%), dahlia mosaic virus (DMV, 62.8%) and CaMV (62.0%) (Supplementary Table S18, Figure 6b). The identity matrix for amino acid sequences showed the highest identity with GPRV for PaCV-1 (64.1%), figwort mosaic virus (FMV, 51.3%) and CaMV (51.4%) (Supplementary Table S19, Figure 6c). On the dendrogram, the sequence of the polymerase region of GPRV clustered with PaCV-1 and RuFDV as well as with strawberry vein banding virus (SVBV) and metaplexis yellow mottle-associated virus (MeYMaV) (Figure 7b,c).

Due to the co-infection of the studied grapevines with several pathogens, including viruses and viroids, it was not possible to associate the presence of GPRV with specific symptoms at this stage of the study.

As demarcation criteria for assigning a new species to the genus *Caulimovirus*, we used the host range and differences in polymerase (RT + RNAse H) nucleotide sequences of more than 20% [80]. In our study, we identified GPRV in grape samples, and the nucleotide sequence of the polymerase gene had an identity with the sequences of other caulimovirus species at the level of 43.9–64.1%. Because both of these two conditions are satisfied, this allows us to suggest that a novel species of the genus *Caulimovirus*, with a tentative name grapevine pararetrovirus (GPRV), was discovered in the Dagestan germplasm collections.

**Figure 8:**
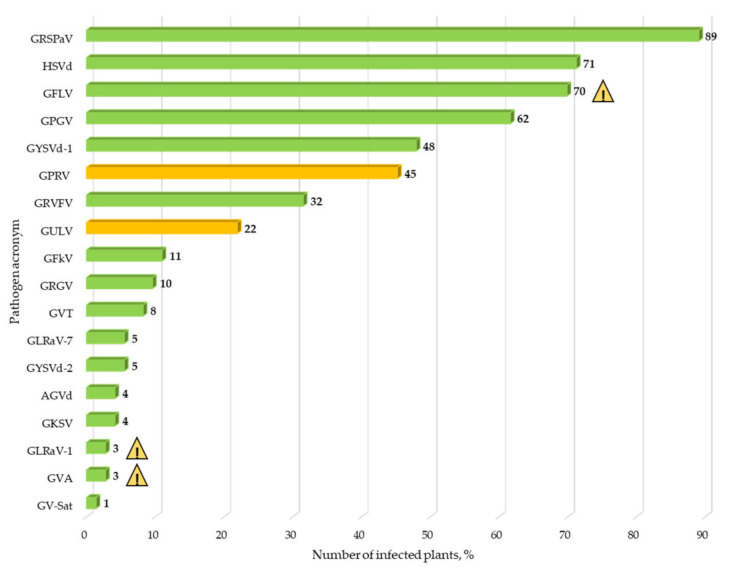
Frequency of spread of grapevine viruses and virus-like organisms in Dagestan ampelographic collections (percentage of the total number of collected samples). Two new viruses are marked orange. Economically significant viruses are shown as 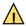.

Collections of grape germplasm are not only a source of valuable traits but are also a place of accumulation of various pathogens, including viruses that affect the production characteristics of new varieties and hybrids. In our study, we selected 73 plants with symptoms of a viral infection at two Dagestan ampelographic collections. More than 50% of them were varieties of local selection (the region of origin is Dagestan) and about 28% were varieties of foreign selection cultivated for a long time at these collections (Supplementary Table S1). Based on bioinformatics analysis of sequencing data and their validation by RT-PCR and RT-qPCR methods, we identified 14 viruses and 4 viroids; 2 viruses, GKSV and GLRaV-7, were detected in Russia for the first time (Figure 8). All samples were characterized by mixed viral infection (from 1 to 11 viruses/viroids per sample) (Supplementary Table S1). The least infected sample of the Bayat Kapy variety was infected with 1 virus (library Q86). The most infected samples (11 pathogens) were varieties of Uzbekistan origin (Vasarga White, library Q65) and Dagestan origin (Maraginskii Black, library Q73).

## 4. Discussion

This study is part of a project to analyze the species diversity of viruses in ampelographic collections in Russia. Previously, we analyzed the virome of the Anapa ampelographic collection [30]. The species compositions of viruses and viroids in the Anapa and Dagestan collections are 50% identical. The most common viruses in these collections are GRSPaV and GPGV; they were detected in 62–98% of samples. In Dagestan samples, GFkV was relatively rare (11% of grapevine accessions) compared with its high frequency of occurrence in the Anapa collection (72% of samples) and in commercial vineyards in Russia (71–79% of samples) [53,54]. In Anapa vines, GLRaV-1, -2, -3 and -4 were detected in 2–13% of samples, while in Dagestan samples, GLRaVs were less represented; GLRaV-1 was found in only 3% of samples and GLRaV-7 was found in 5%. Vitiviruses are also less represented in the Dagestan collections; of all the species of this genus, only GVA was found in 3% of the samples compared with GVA, grapevine virus B (GVB) and grapevine virus F (GVF) in 2–9% of the samples. GVT that belongs to the genus *Foveavirus* was identified in 8% of the Dagestan samples and 26% of the Anapa samples. Several viruses had a similar distribution in the Dagestan and Anapa collections: GRGV (10 and 6%, respectively), GRVFV (33% and 49%, respectively) and GV-Sat (1 and 4%, respectively). Among viroids, the most common were GYSVd-1 and HSVd (48% and 71% in the Dagestan samples compared to 98% for each viroid in the Anapa samples), whereas GYSVd-2 was not found in the Anapa samples at all. AGVd was present in 2–4% of the samples in the collections of the two regions. In the germplasm collections of Dagestan, a wide distribution of an economically harmful pathogen was observed (GFLV (70%)), which was not found in Anapa samples. A number of GFLV isolates contained satellite RNA, which was detected by us for the first time in this study. The different species compositions of viruses and viroids is most likely associated with the numbers and different sources of varieties and hybrids in collections since in the Dagestan collections, most of them are represented by varieties of local selection and these collections have a smaller number of samples, while the Anapa collection contains about 5000 samples imported from all over the world [83].

In this study, we have detected two novel grape-infecting viruses in the Dagestan samples: (+) ssRNA GULV (genus *Umbravirus*) and dsDNA pararetrovirus GPRV (genus *Caulimovirus*). In an earlier analysis of the samples from the Anapa collection, we identified a GULV contig, but we were unable to obtain a longer nucleotide sequence of the genome of this virus in that study. In this work, we assembled complete or nearly complete genomes from three GULV isolates. It is known that umbraviruses do not encode a coat protein but use the one from helper viruses. In other umbraviruses, this role is played by species from the family Luteoviridae [78]. However, representatives of this genus have not been found in grapes. Therefore, the search for helper species is the subject of future research on this virus. The distribution of GULV in the Dagestan and Anapa collections is 22% and 6%, respectively.

Another novel virus we discovered, GPRV, is the first assembled caulimovirus of grapes. It is interesting that in the bioinformatics analysis of this virus, we identified contigs using the Geneious assembler but not using SPAdes. This, as in our previous studies [30,54], confirms the need to analyze data by various methods (e.g., the use of two assemblers along with mapping reads to known reference sequences of grapevine viruses and viroids) for the most complete description of the virome.

In the future, the viruses we discovered require a more detailed study in terms of their biology, genome expression and impact. Considering the high harmfulness of representatives of the genus *Caulimovirus* (for example, CaMV [84,85,86]) and *Umbravirus* (for example, GRV [87]), it is necessary to determine the pathogenicity of GPRV and GULV for grapes. Phytosanitary monitoring will allow the establishment of a correlation between the manifestation of symptoms and the presence of these viruses in the plant as well as possible diseases associated with them. Assessment of the economic harm of GPRV or GULV infections, as well as the study of their distribution in vineyards (including wild grapevines) in Russia, will be the subject of our future research.

This study confirmed a high infection rate of Russian grape germplasms with viruses. Because the samples that we analyzed are the source material in the selection programs and are cultivated in the same vineyards with new varieties and hybrids, the transmission of viruses, including economically harmful ones, to valuable genetic material is inevitable. Therefore, the results of our work should be used for integrated grape protection and protective programs aimed at limiting the spread of vectors, the gradual sanitation of the collections by biotechnological methods and their re-laying in new places to limit the spread of viruses and viroids.

## Figures and Tables

**Table 1:** Analysis of contigs of a putative new species of dsDNA grapevine-associated virus.

Library	tblastx of Contigs			blastn (or blastx for Q73) of Contigs by NCBI Tool			
	Virus Name	Contig E-Value	Contig Length, bp	Closest Isolate	Percent Identity, %	Query Cover, %	GenBank Identifier
Q24	RuFDV *	–51	461	PaCV-1	70.26%	91%	OL472131.1
				CaMV	68.49%	86%	AB863188.1
	SMMP	–38	342	DMV	67.93%	84%	HQ416677.1
				AnBSV	66.67%	92%	NC_043523.1
Q50	CaMV	–36	432	DMV	71.08%	47%	EF203675.1
				CaMV	70.26%	61%	LC632935.1
				HRLV	68.34%	46%	JX429923.1
				MMV	69.15%	43%	AF454635.1
				FMV	67.65%	47%	X06166.1
Q52	CaMV	–58	431	PaCV-1	74.84%	72%	OL472131.1
				CaMV	70.39%	89%	KY810770.1
Q58	RuFDV	–37	318	PaCV-1	72.77%	73%	OL472131.1
				AMMV	68.42%	82%	KR080327.1
				FMV	66.41%	81%	X06166.1
				AnBSV	66.67%	77%	NC_043523.1
Q73	RuFDV	–16	265	PaCV-1	68.09%	53%	UTQ50644.1
				RuFDV	64.81%	61%	YP_002519387.1
				CaMV	64.81%	61%	AB863188.1
				DMV	61.70%	53%	AEB54984.1
				LLDV	63.64%	49%	YP_001931961.1
Q84	RuFDV	–30	299	PaCV-1	69.85%	66%	OL472131.1
				FMV	70.76%	57%	X06166.1

* Genus *Ruflodivirus*: RuFDV (rudbeckia flower distortion virus); genus *Caulimovirus*: CaMV (cauliflower mosaic virus), SMMP (soybean mild mottle pararetrovirus), DMV (dahlia mosaic virus), AnBSV (Angelica bushy stunt virus), HRLV (horseradish latent virus), MMV (Mirabilis mosaic virus), FMV (figwort mosaic virus), AMMV (Atractylodes mild mottle virus), LLDV (Lamium leaf distortion virus); unclassified *Caulimovirus*: PaCV-1 (plant associated caulimovirus 1).

## Data Availability

Representative sequences were deposited in GenBank under the accession numbers: OP727271, OP885187–OP885211, OP885212–OP885327, OP886234–OP886421.

## Supplementary Materials

The following supporting information can be downloaded at: https://www.mdpi.com/article/10.3390/v14122623/s1, Figure S1: Neighbor-joining tree showing the distribution of complete nucleotide sequences RNA1 of Russian grapevine fanleaf virus (GFLV) isolates and world isolates; Figure S2: Neighbor-joining tree showing the distribution of complete nucleotide sequences RNA2 of Russian grapevine fanleaf virus (GFLV) isolates and world isolates; Figure S3: Neighbor-joining tree showing the distribution of complete genome nucleotide sequences of Russian grapevine fanleaf virus satellite RNA (GFLV sat) isolate and world GFLV sat and arabis mosaic virus satellite RNA (ArMV sat) isolates; Figure S4: Neighbor-joining tree showing the distribution of complete genome nucleotide sequences of Russian grapevine rupestris stem pitting-associated virus (GRSPaV) isolates and world isolates; Figure S5: Neighbor-joining tree showing the distribution of complete genome nucleotide sequences of Russian grapevine Pinot gris virus (GPGV) isolate and world isolates; Figure S6: Alignment of MP/CP nucleotide sequences and movement proteins (MP) of Russian grapevine Pinot gris virus (GPGV) isolates; Figure S7: Neighbor-joining tree showing the distribution of complete genome nucleotide sequences of Russian grapevine virus A (GVA) isolates and world isolates; Figure S8: Neighbor-joining tree showing the distribution of complete genome nucleotide sequences of Russian grapevine virus T (GVT) isolates and world isolates; Figure S9: Neighbor-joining tree showing the distribution of complete genome nucleotide sequences of Russian grapevine Kizil Sapak virus (GKSV) isolates and world isolates; Figure S10: Neighbor-joining tree showing the distribution of complete genome nucleotide sequences of Russian grapevine leafroll-associated virus 1 (GLRaV-1) isolate and world isolates; Figure S11: Neighbor-joining tree showing the distribution of coat protein nucleotide sequences of Russian grapevine leafroll-associated virus 1 (GLRaV-1) isolate and world isolates; Figure S12: Neighbor-joining tree showing the distribution of complete genome nucleotide sequences of Russian grapevine leafroll-associated virus 7 (GLRaV-7) isolates and world isolates; Figure S13: Neighbor-joining tree showing the distribution of complete genome nucleotide sequences of Russian grapevine satellite virus (GV-Sat) isolate and world isolates; Figure S14: Neighbor-joining tree showing the distribution of complete genome nucleotide sequences of Russian hop stunt viroid (HSVd) isolates and world isolates; Figure S15: Neighbor-joining tree showing the distribution of complete genome nucleotide sequences of Russian grapevine yellow speckle viroid 1 (GYSVd-1) isolates and world isolates; Figure S16: Neighbor-joining tree showing the distribution of complete genome nucleotide sequences of Russian grapevine yellow speckle viroid 2 (GYSVd-2) isolates and world isolates; Figure S17: Neighbor-joining tree showing the distribution of complete genome nucleotide sequences of Russian Australian grapevine viroid (AGVd) isolate and world isolates; Table S1: Basic information of selected grape samples and viral infection; Table S2: Complete sequences of viruses and viroids uploaded into GenBank with their identifier; Table S3: List of the RT-PCR primers used for virus detection [30,54,60,76,88,89,90,91,92,93,94,95,96,97,98,99]; Table S4: List of sequences after Sanger sequencing submitted to the GenBank; Table S5: List of the RT-qPCR primers used for virus detection and calibration curve parameters for simplex RT-qPCR [100,101]; Table S6: List of representative umbraviruses and umbra-like viruses; Table S7: List of representative badnaviruses, caulimoviruses, unclassified caulimoviruses and ruflodivirus; Table S8: Initial data of complete or almost complete genomes for phylogenetic analysis; Table S9: Representative virus isolates used for group definition [30,61,102,103,104,105]; Table S10: Number of reads and contigs obtained for each library after sequencing by Illumina NovaSeq 6000 Sequencing System; Table S11: Bioinformatics analysis and its validation; Table S12: Bioinformatics analysis, mapping of preprocessed reads to closest genome; Table S13: Viruses detected in GV-Sat positive samples; Table S14: The pairwise identity matrix of complete genomes of GULV and 31 representatives of the genus Umbravirus and umbra-like viruses; Table S15: The pairwise identity matrix of ORF2 (RdRp) nucleotide sequences of GULV and 20 representatives of the genus *Umbravirus* and umbra-like viruses; Table S16: The pairwise identity matrix of ORF2 (RdRp) amino acid sequences of GULV and 20 representatives of the genus *Umbravirus* and umbra-like viruses; Table S17: The pairwise identity matrix of complete genomes of GPRV and 21 representatives of the genus *Badnavirus*, *Ruflodivirus*, *Caulimovirus* and unclassified *Caulimovirus*; Table S18: The pairwise identity matrix of polymerase region nucleotide sequences of GPRV and 18 representatives of the genus *Ruflodivirus*, *Caulimovirus* and unclassified *Caulimovirus*; Table S19: The pairwise identity matrix of polymerase region amino acid sequences of GPRV and 18 representatives of the genus *Ruflodivirus*, *Caulimovirus* and unclassified *Caulimovirus*.
