# Supplementary material for: Metagenomic Analysis of Ampelographic Collections of Dagestan Revealed the Presence of Two Novel Grapevine Viruses
Source: Viruses. 2022 Nov 24;14(12):2623. doi: 10.3390/v14122623 (PMC9781968; doi:10.3390/v14122623)
Supplement: Supplementary file 1 [file viruses-14-02623-s001.zip › Supplementary Firures.pdf]

Metagenomic Analysis of Ampelographic Collections of Dagestan Revealed the Presence of Two Novel Grapevine Viruses

Darya Shvets, Kirill Sandomirsky, Elena Porotikova and Svetlana Vinogradova *

Skryabin Institute of Bioengineering, Research Center of Biotechnology of the Russian Academy of Sciences, Leninsky Prospect, 33, Build. 2, 119071 Moscow, Russia
* Correspondence: coatprotein@bk.ru

Supplementary Materials

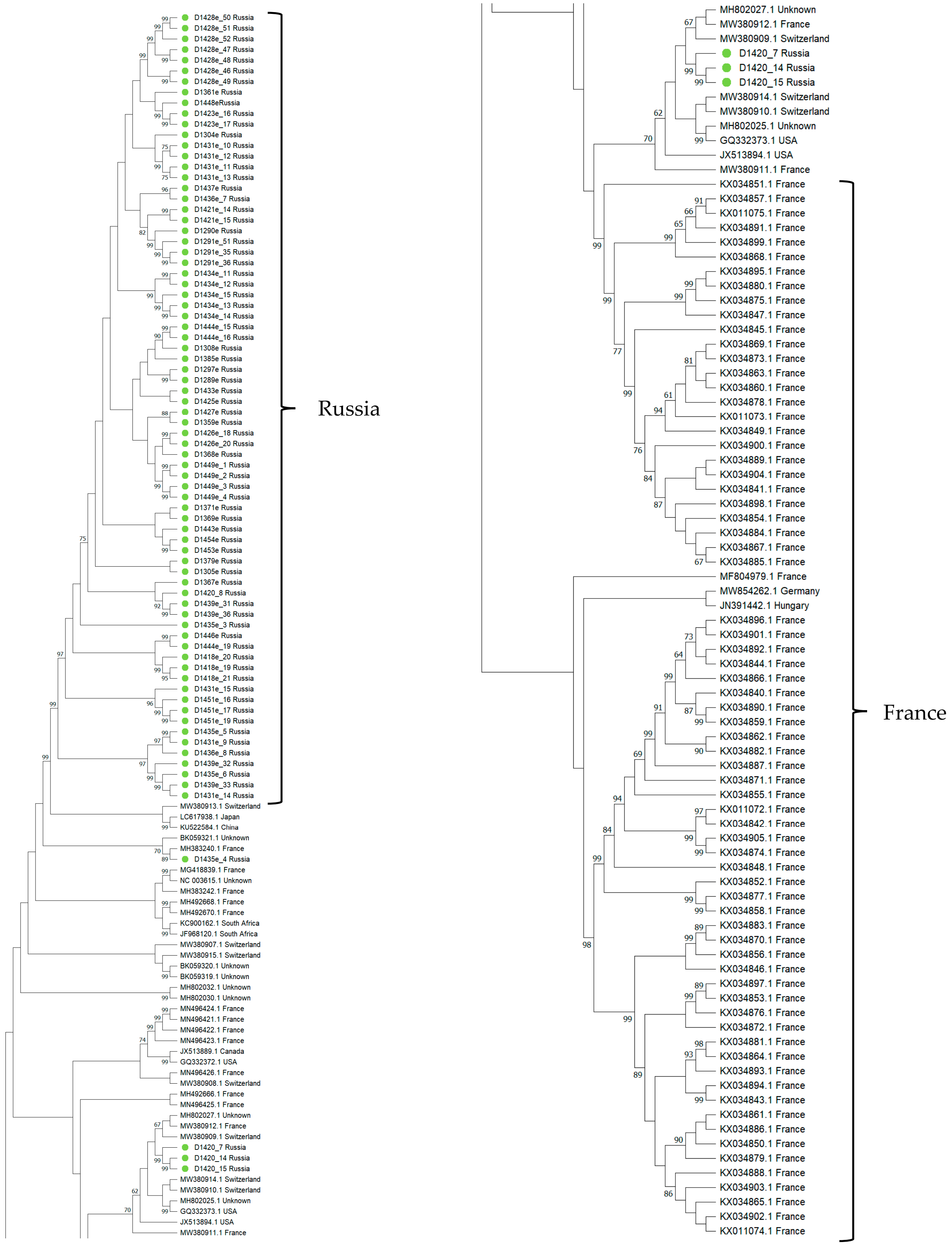

(a) (b)

Supplementary Figure S1 (a, b). Neighbor-joining tree showing the distribution of complete nucleotide sequences RNA1 of Russian grapevine fanleaf virus (GFLV) isolates (●) and world isolates. Bootstrap values >60% (1000 bootstrap replicates) are shown.

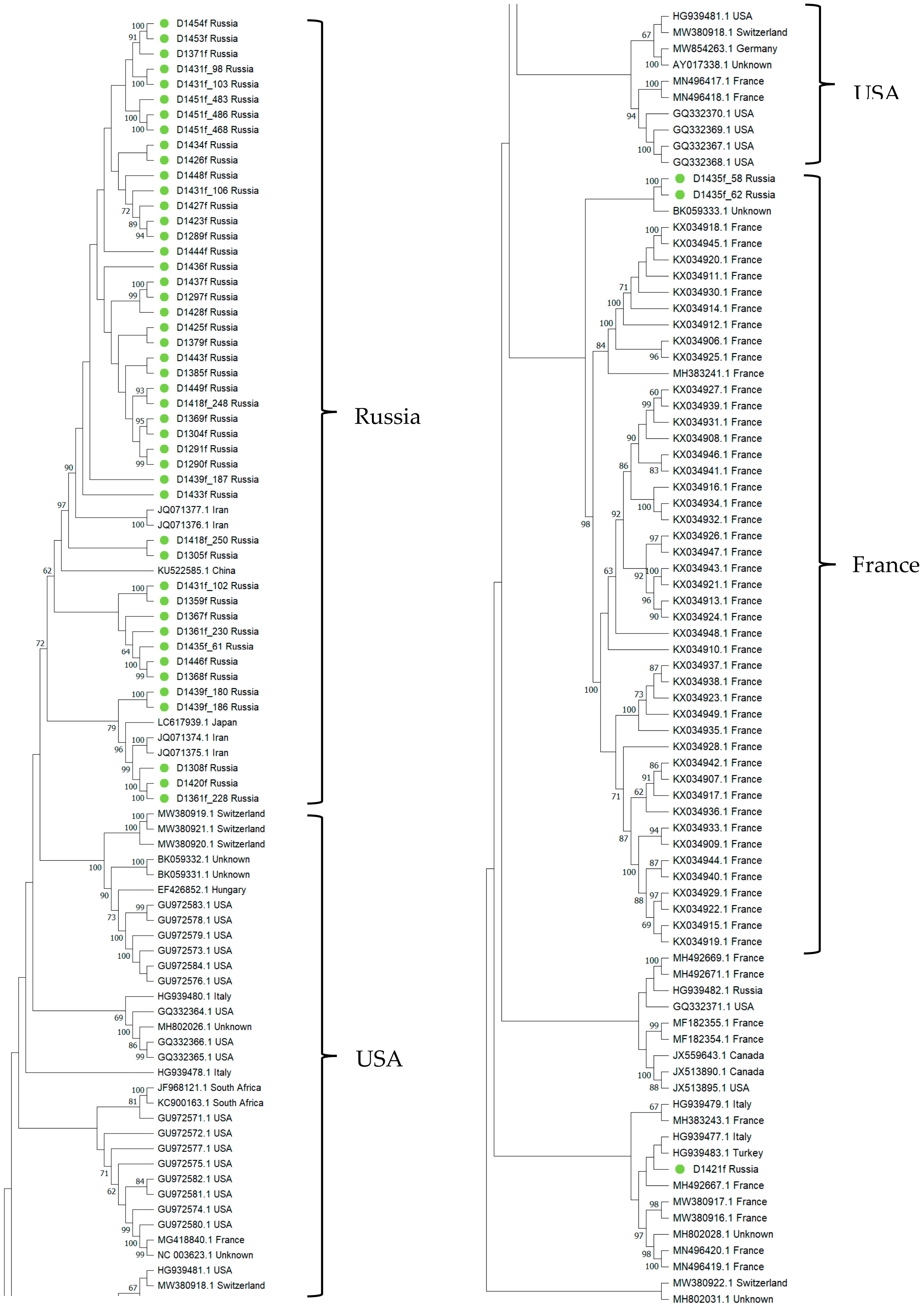

(a) **(b)**

Supplementary Figure S2 (a, b). Neighbor-joining tree showing the distribution of complete nucleotide sequences RNA2 of Russian grapevine fanleaf virus (GFLV) isolates (●) and world isolates. Bootstrap values >60% (1000 bootstrap replicates) are shown.

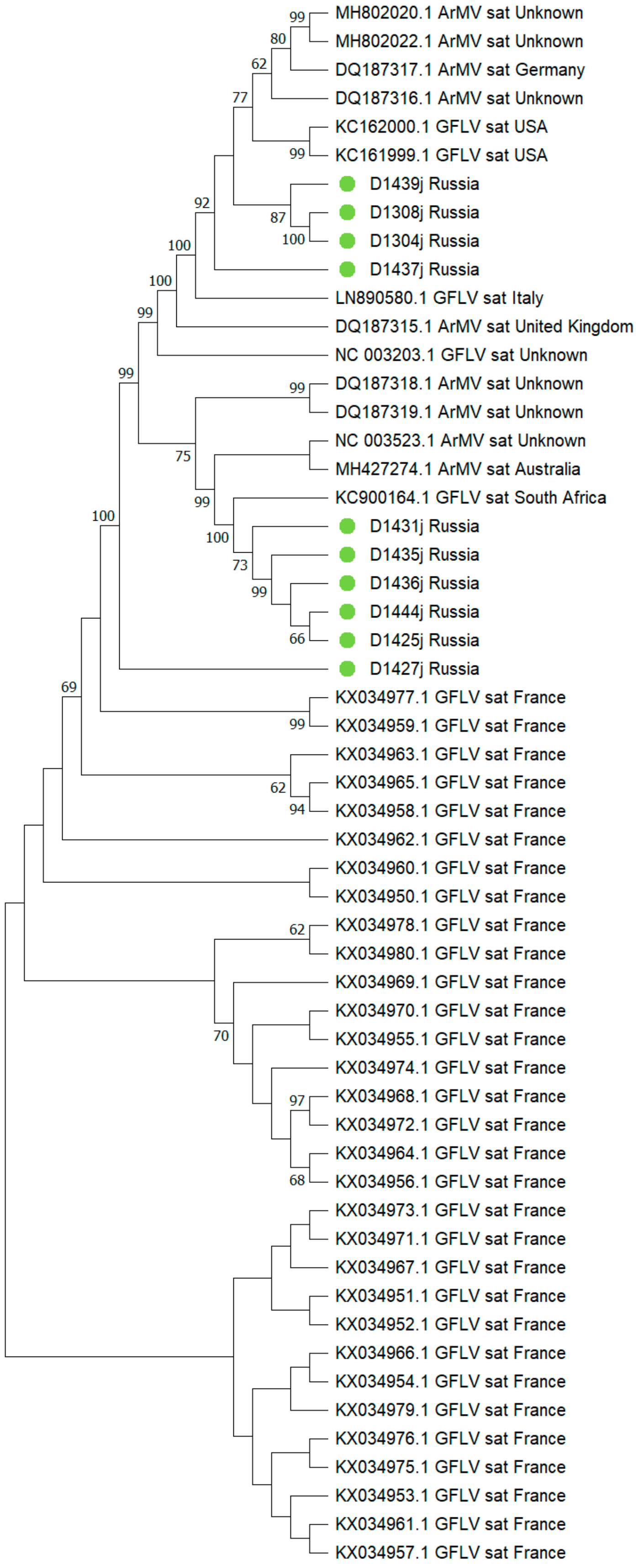

Supplementary Figure S3. Neighbor-joining tree showing the distribution of complete genome nucleotide sequences of Russian grapevine fanleaf virus satellite RNA (GFLV sat) isolate (●) and world GFLV sat and Arabis mosaic virus satellite RNA (ArMV sat) isolates. Bootstrap values >60% (1000 bootstrap replicates) are shown.

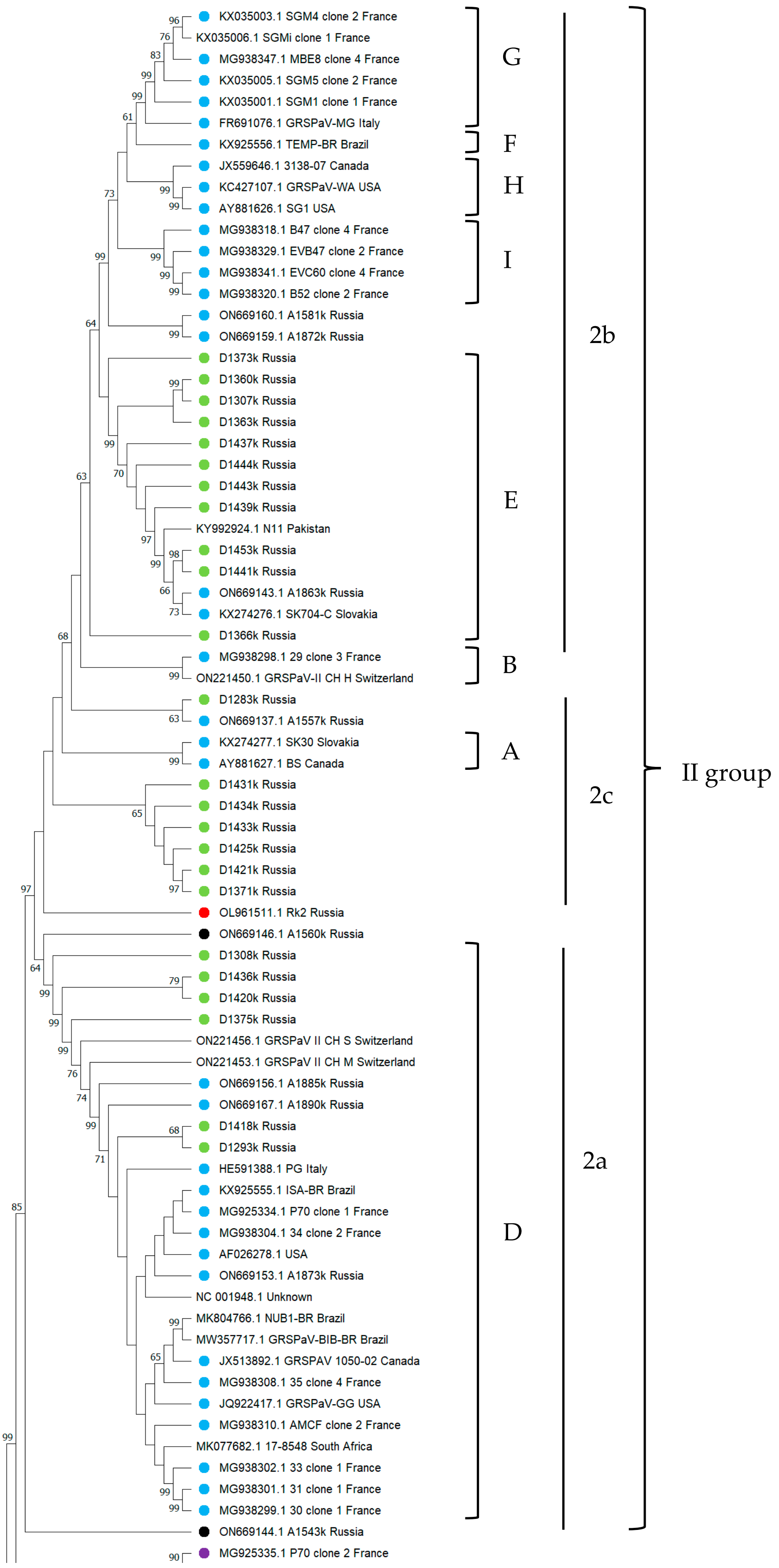
KX035003.1 SGM4 clone 2 France
KX035006.1 SGMi clone 1 France
MG938347.1 MBE8 clone 4 France
KX035005.1 SGM5 clone 2 France
KX035001.1 SGM1 clone 1 France
FR691076.1 GRSPaV-MG Italy
KX925556.1 TEMP-BR Brazil
JX559646.1 3138-07 Canada
KC427107.1 GRSPaV-WA USA
AY881626.1 SG1 USA
MG938318.1 B47 clone 4 France
MG938329.1 EVB47 clone 2 France
MG938341.1 EVC60 clone 4 France
MG938320.1 B52 clone 2 France
ON669160.1 A1581k Russia
ON669159.1 A1872k Russia
D1373k Russia
D1360k Russia
D1307k Russia
D1363k Russia
D1437k Russia
D1444k Russia
D1443k Russia
D1439k Russia
KY992924.1 N11 Pakistan
D1453k Russia
D1441k Russia
ON669143.1 A1863k Russia
KX274276.1 SK704-C Slovakia
D1366k Russia
MG938298.1 29 clone 3 France
ON221450.1 GRSPaV-II CH H Switzerland
D1283k Russia
ON669137.1 A1557k Russia
KX274277.1 SK30 Slovakia
AY881627.1 BS Canada
D1431k Russia
D1434k Russia
D1433k Russia
D1425k Russia
D1421k Russia
D1371k Russia
OL961511.1 Rk2 Russia
ON669146.1 A1560k Russia
D1308k Russia
D1436k Russia
D1420k Russia
D1375k Russia
ON221456.1 GRSPaV II CH S Switzerland
ON221453.1 GRSPaV II CH M Switzerland
ON669156.1 A1885k Russia
ON669167.1 A1890k Russia
D1418k Russia
D1293k Russia
HE591388.1 PG Italy
KX925555.1 ISA-BR Brazil
MG925334.1 P70 clone 1 France
MG938304.1 34 clone 2 France
AF026278.1 USA
ON669153.1 A1873k Russia
NC 001948.1 Unknown
MK804766.1 NUB1-BR Brazil
MW357717.1 GRSPaV-BIB-BR Brazil
JX513892.1 GRSPAV 1050-02 Canada
MG938308.1 35 clone 4 France
JQ922417.1 GRSPaV-GG USA
MG938310.1 AMCF clone 2 France
MK077682.1 17-8548 South Africa
MG938302.1 33 clone 1 France
MG938301.1 31 clone 1 France
MG938299.1 30 clone 1 France
ON669144.1 A1543k Russia
MG925335.1 P70 clone 2 France
G
F
H
I
E
B
A
D
2b
2c
2a
II group

(a)

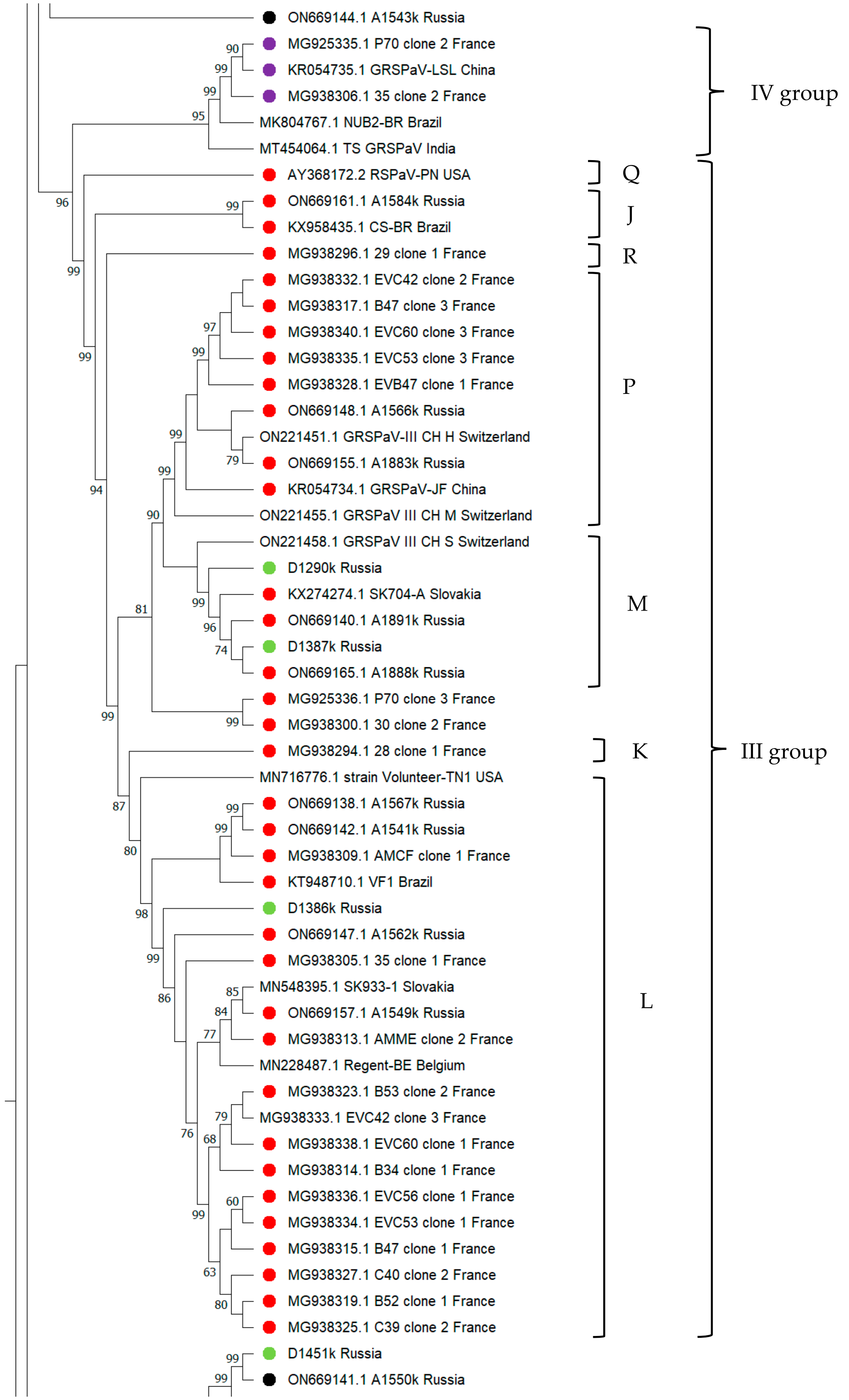
ON669144.1 A1543k Russia
MG925335.1 P70 clone 2 France
KR054735.1 GRSPaV-LSL China
MG938306.1 35 clone 2 France
MK804767.1 NUB2-BR Brazil
MT454064.1 TS GRSPaV India
AY368172.2 RSPaV-PN USA
ON669161.1 A1584k Russia
KX958435.1 CS-BR Brazil
MG938296.1 29 clone 1 France
MG938332.1 EVC42 clone 2 France
MG938317.1 B47 clone 3 France
MG938340.1 EVC60 clone 3 France
MG938335.1 EVC53 clone 3 France
MG938328.1 EVB47 clone 1 France
ON669148.1 A1566k Russia
ON221451.1 GRSPaV-III CH H Switzerland
ON669155.1 A1883k Russia
KR054734.1 GRSPaV-JF China
ON221455.1 GRSPaV III CH M Switzerland
ON221458.1 GRSPaV III CH S Switzerland
D1290k Russia
KX274274.1 SK704-A Slovakia
ON669140.1 A1891k Russia
D1387k Russia
ON669165.1 A1888k Russia
MG925336.1 P70 clone 3 France
MG938300.1 30 clone 2 France
MG938294.1 28 clone 1 France
MN716776.1 strain Volunteer-TN1 USA
ON669138.1 A1567k Russia
ON669142.1 A1541k Russia
MG938309.1 AMCF clone 1 France
KT948710.1 VF1 Brazil
D1386k Russia
ON669147.1 A1562k Russia
MG938305.1 35 clone 1 France
MN548395.1 SK933-1 Slovakia
ON669157.1 A1549k Russia
MG938313.1 AMME clone 2 France
MN228487.1 Regent-BE Belgium
MG938323.1 B53 clone 2 France
MG938333.1 EVC42 clone 3 France
MG938338.1 EVC60 clone 1 France
MG938314.1 B34 clone 1 France
MG938336.1 EVC56 clone 1 France
MG938334.1 EVC53 clone 1 France
MG938315.1 B47 clone 1 France
MG938327.1 C40 clone 2 France
MG938319.1 B52 clone 1 France
MG938325.1 C39 clone 2 France
D1451k Russia
ON669141.1 A1550k Russia
IV group
Q
J
R
P
M
K
L
III group

(b)

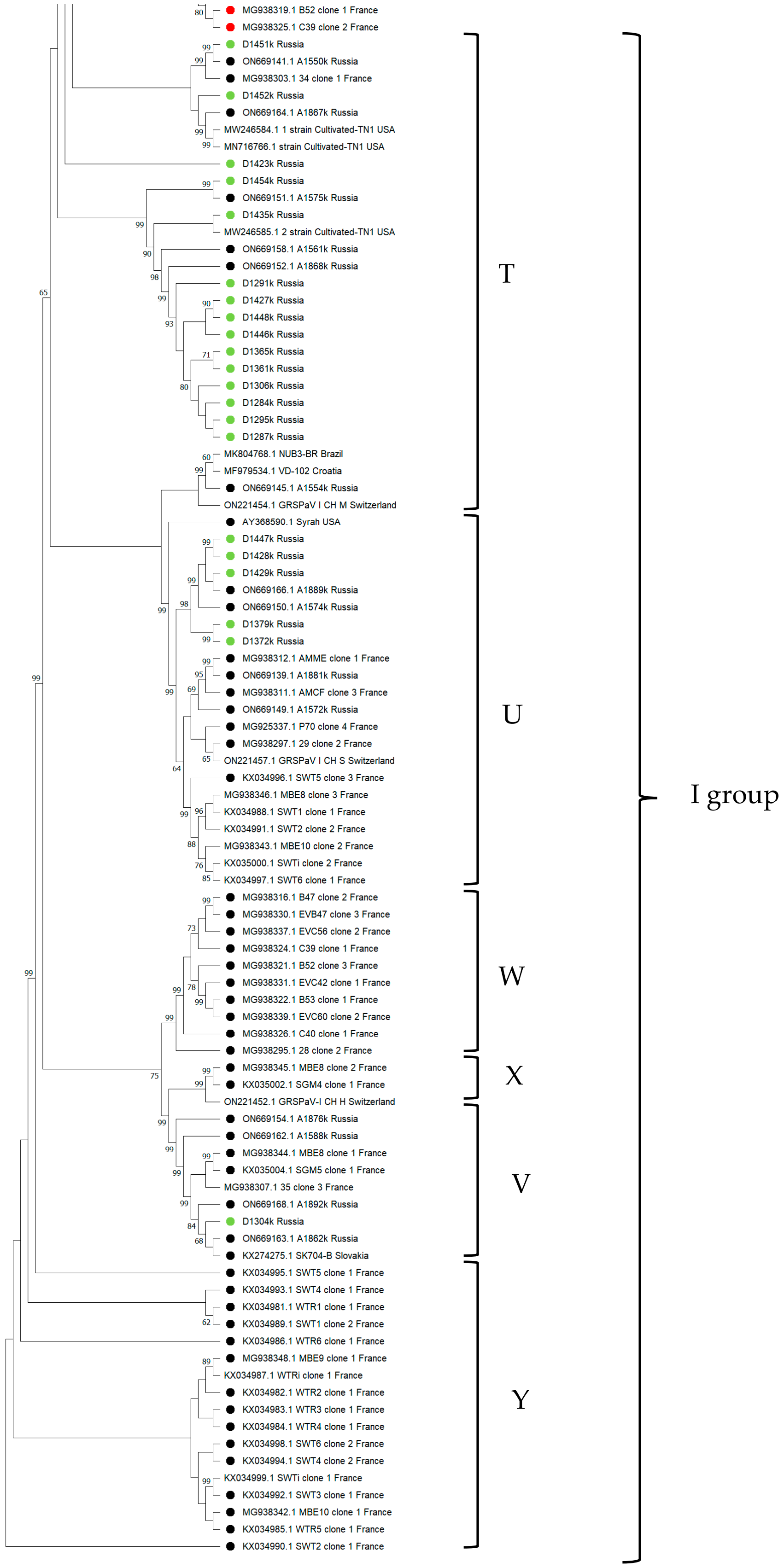

(c)

Supplementary Figure S4 (a-c). Neighbor-joining tree showing the distribution of complete genome nucleotide sequences of Russian grapevine rupestris stem pitting-associated virus (GRSPaV) isolates (●) and world isolates. I, II, III and IV molecular groups are indicated as (●), (●), (●) and (●), respectively. Bootstrap values >60% (1000 bootstrap replicates) are shown.

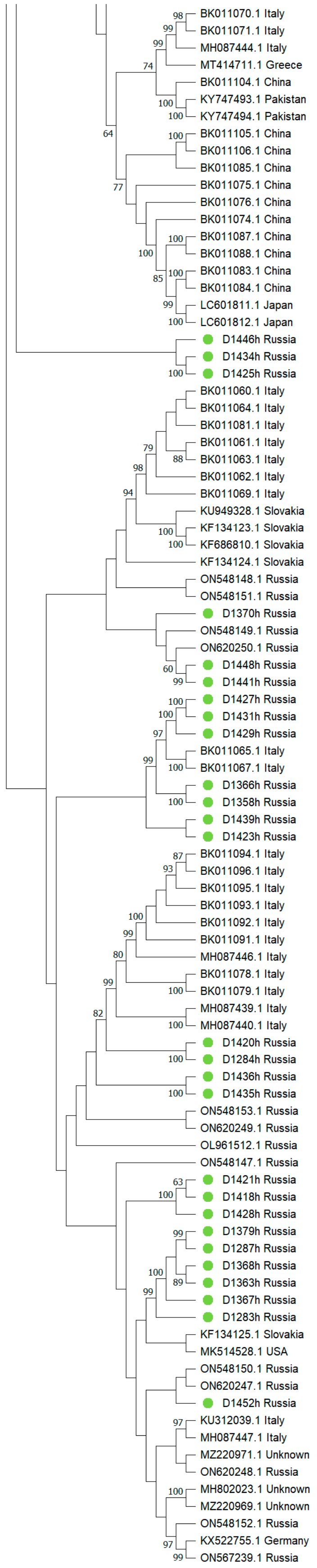

Supplementary Figure S5. Neighbor-joining tree showing the distribution of complete genome nucleotide sequences of Russian grapevine Pinot gris virus (GPGV) isolate (●) and world isolates. Bootstrap values >60% (1000 bootstrap replicates) are shown.

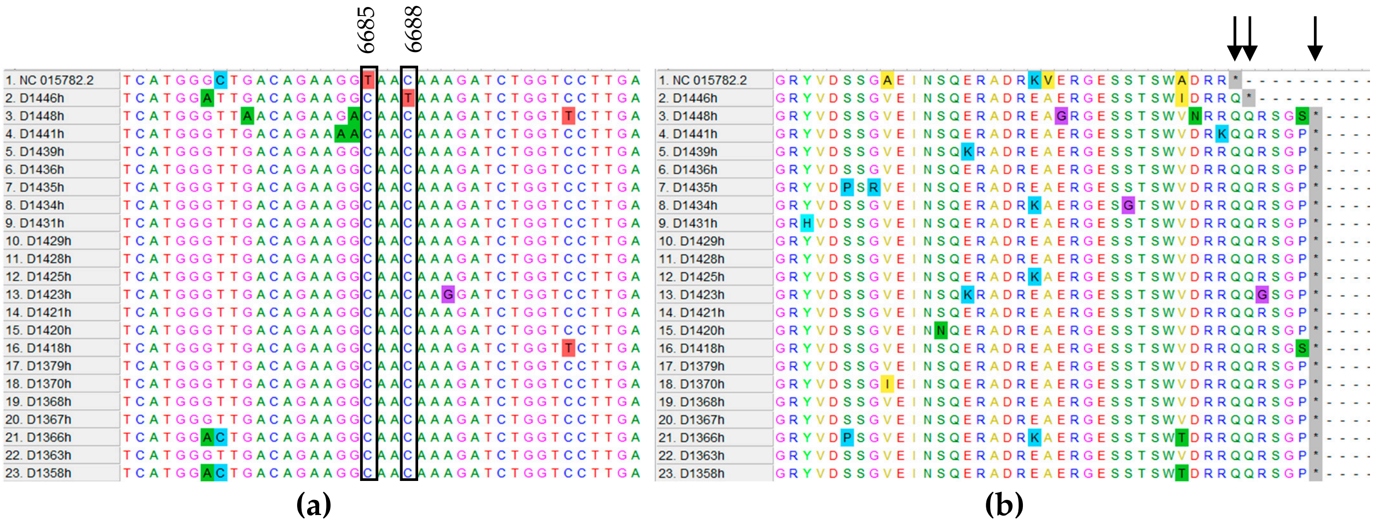

Supplementary Figure S6 (a, b). Alignment of (**a**) MP/CP nucleotide sequences; (**b**) movement proteins (MP) of Russian grapevine Pinot gris virus (GPGV) isolates and reference isolates (NC_015782.2). 6685 and 6688 T/C polymorphisms are marked in boxes. The stop codons are indicated by gray stars.

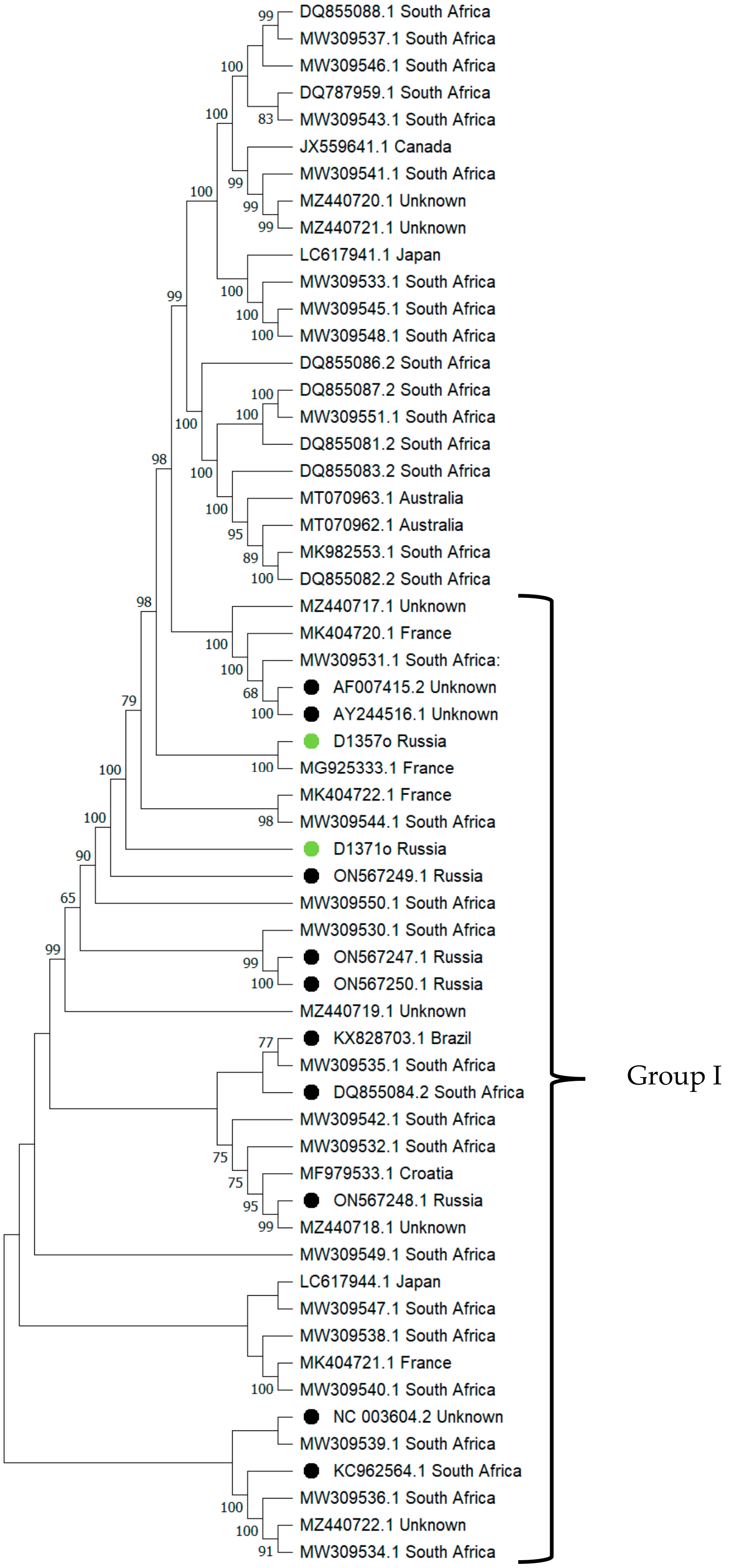

Supplementary Figure S7. Neighbor-joining tree showing the distribution of complete genome nucleotide sequences of Russian grapevine virus A (GVA) isolates (●) and world isolates. Representative isolates from I molecular group are indicated as (●). Bootstrap values >60% (1000 bootstrap replicates) are shown.

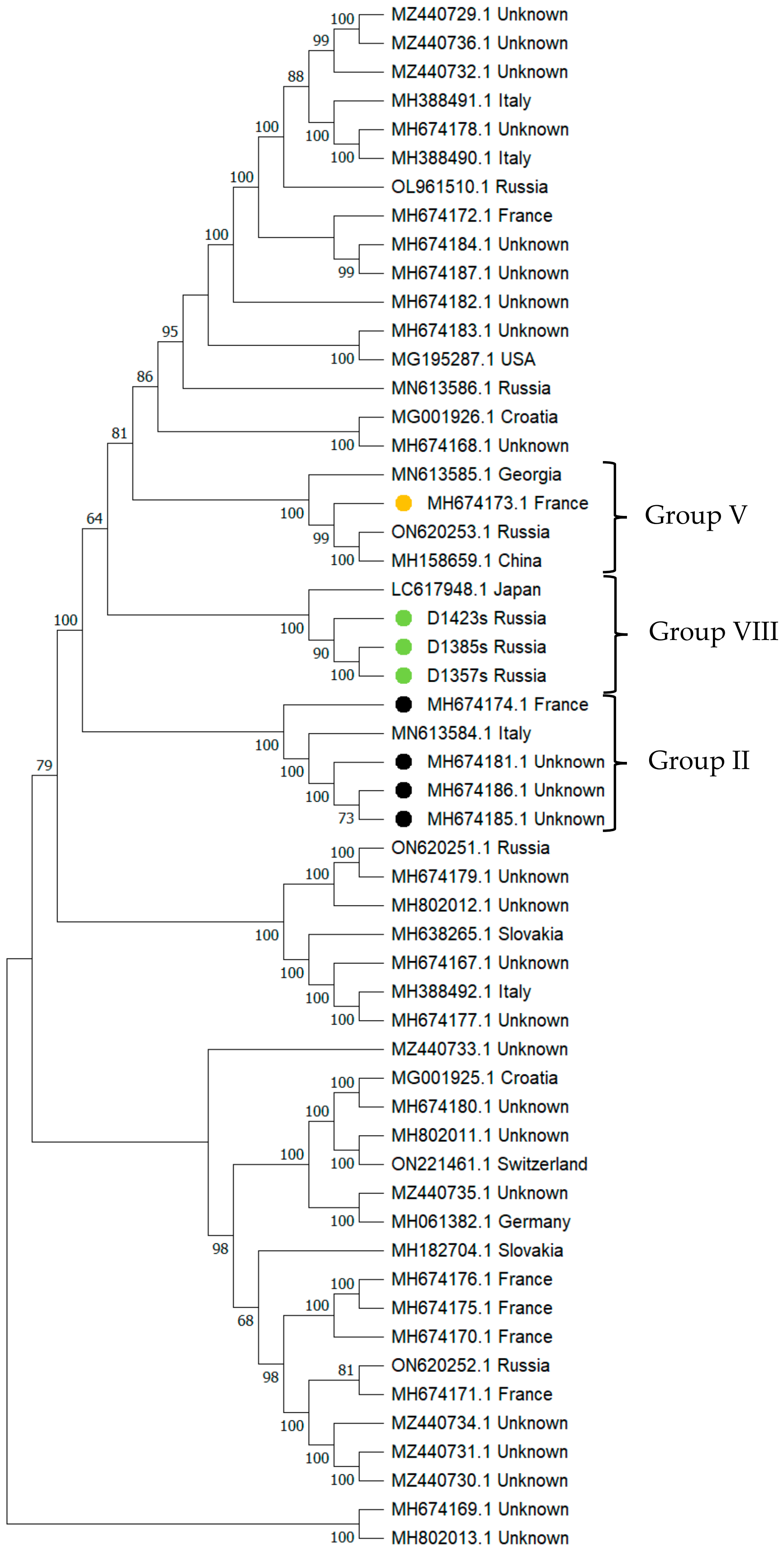

Supplementary Figure S8. Neighbor-joining tree showing the distribution of complete genome nucleotide sequences of Russian grapevine virus T (GVT) isolates (●) and world isolates. II and V molecular groups are indicated as (●) and (●), respectively. Bootstrap values >60% (1000 bootstrap replicates) are shown.

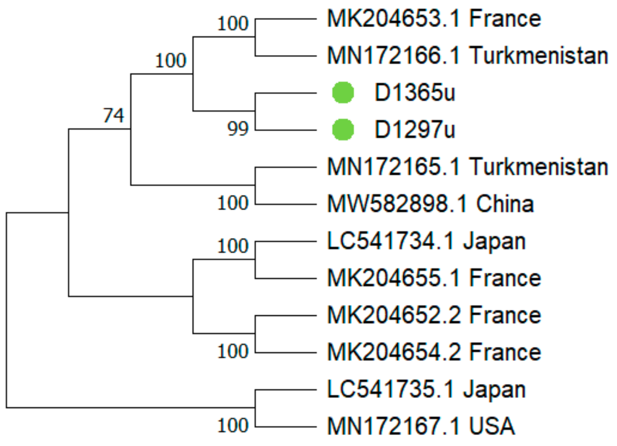

Supplementary Figure S9. Neighbor-joining tree showing the distribution of complete genome nucleotide sequences of Russian grapevine Kizil Sapak virus (GKSV) isolates (●) and world isolates. Bootstrap values >60% (1000 bootstrap replicates) are shown.

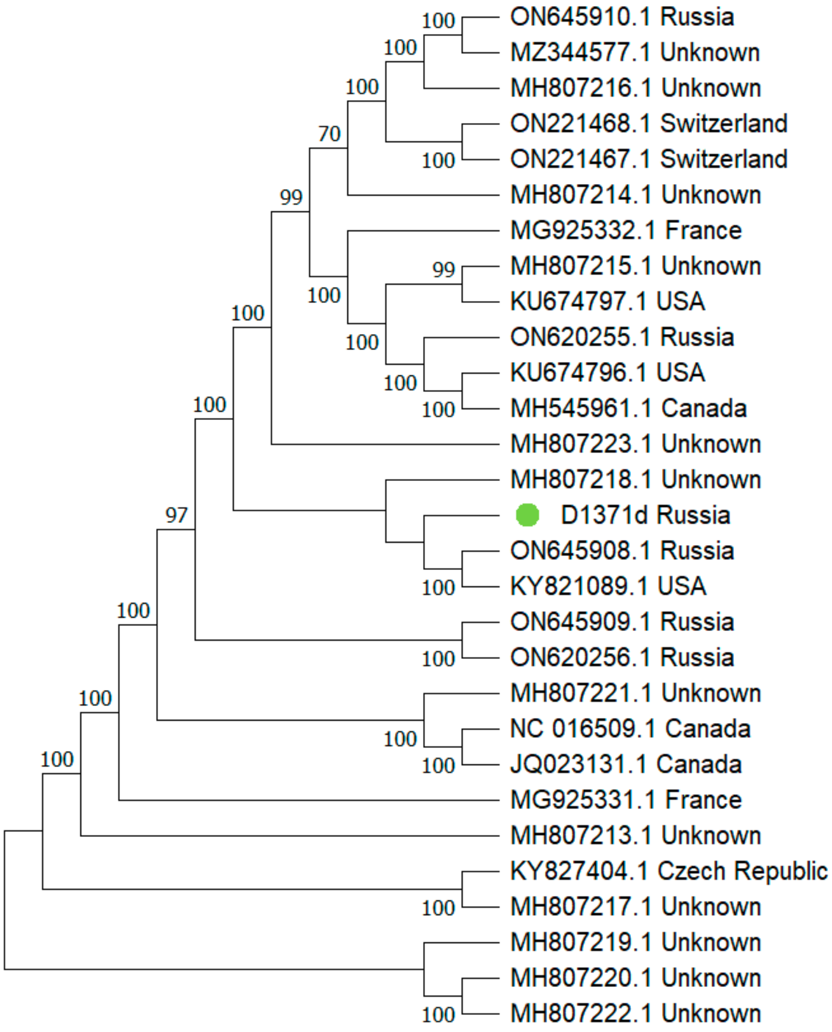

Supplementary Figure S10. Neighbor-joining tree showing the distribution of complete genome nucleotide sequences of Russian grapevine leafroll-associated virus 1 (GLRaV-1) isolate (●) and world isolates. Bootstrap values >60% (1000 bootstrap replicates) are shown.

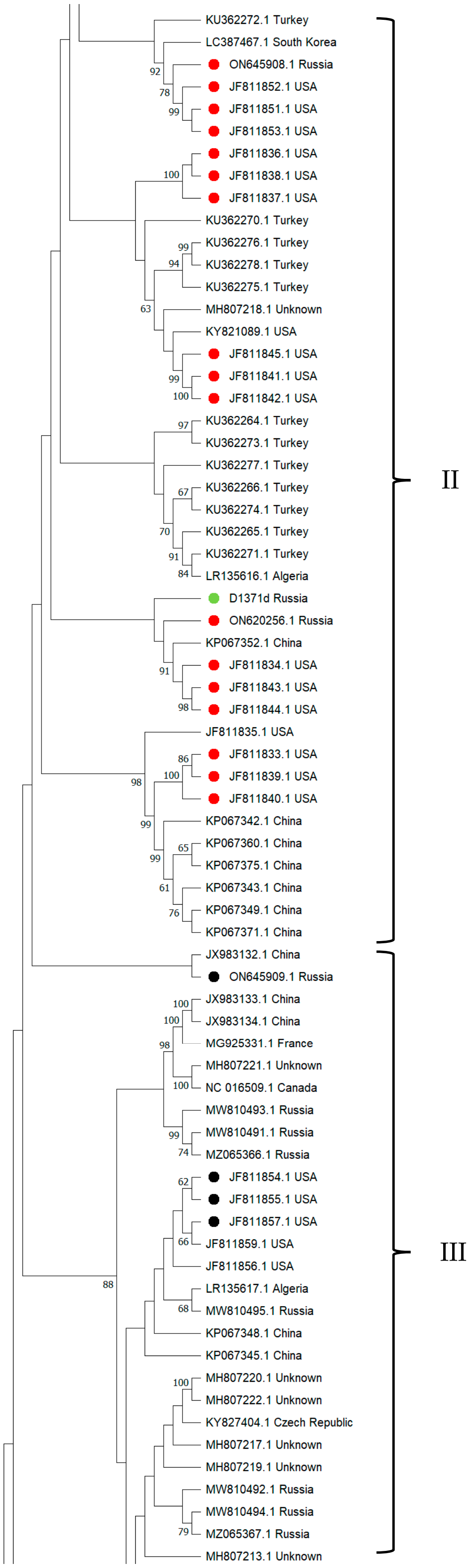

Supplementary Figure S11. Neighbor-joining tree showing the distribution of coat protein nucleotide sequences of Russian grapevine leafroll-associated virus 1 (GLRaV-1) isolate (●) and world isolates. II and III molecular groups are indicated as (●) and (●), respectively. Bootstrap values >60% (1000 bootstrap replicates) are shown.

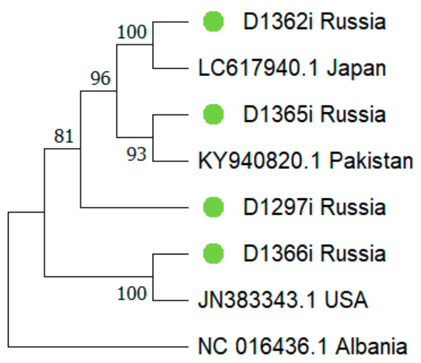

Supplementary Figure S12. Neighbor-joining tree showing the distribution of complete genome nucleotide sequences of Russian grapevine leafroll-associated virus 7 (GLRaV-7) isolates (●) and world isolates. Bootstrap values >60% (1000 bootstrap replicates) are shown.

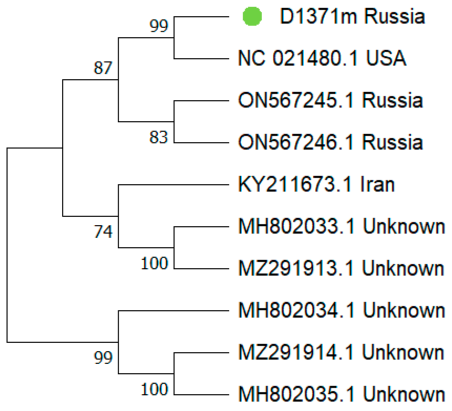

Supplementary Figure S13. Neighbor-joining tree showing the distribution of complete genome nucleotide sequences of Russian grapevine satellite virus (GV-Sat) isolate (●) and world isolates. Bootstrap values >60% (1000 bootstrap replicates) are shown.

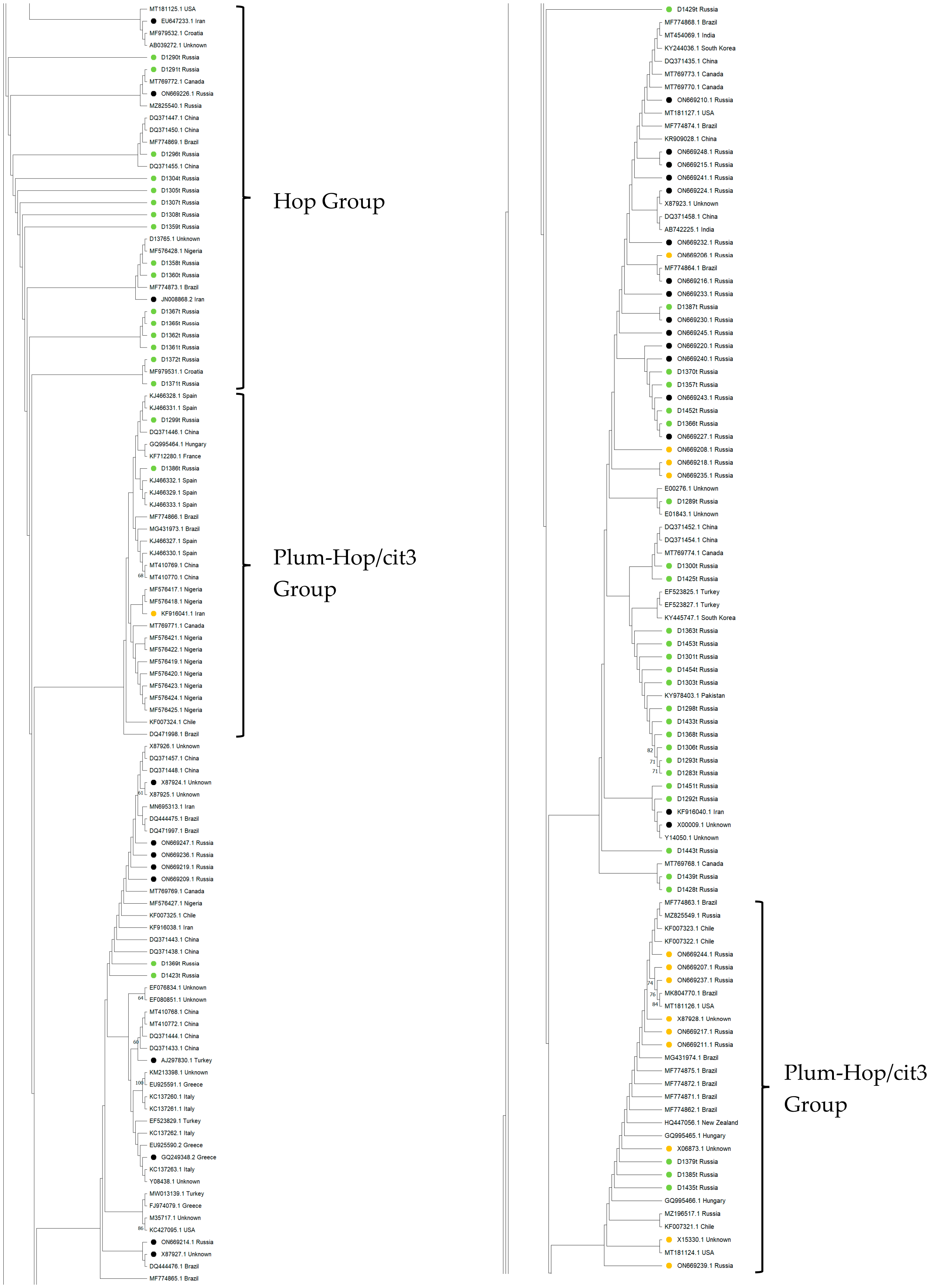

(a) **(b)**

Supplementary Figure S14 (a, b). Neighbor-joining tree showing the distribution of complete genome nucleotide sequences of Russian hop stunt viroid (HSVd) isolates (🟢) and world isolates. Hop and Plum-Hop/cit3 molecular groups are indicated as (⚫) and (🟡), respectively. Bootstrap values >60% (1000 bootstrap replicates) are shown.

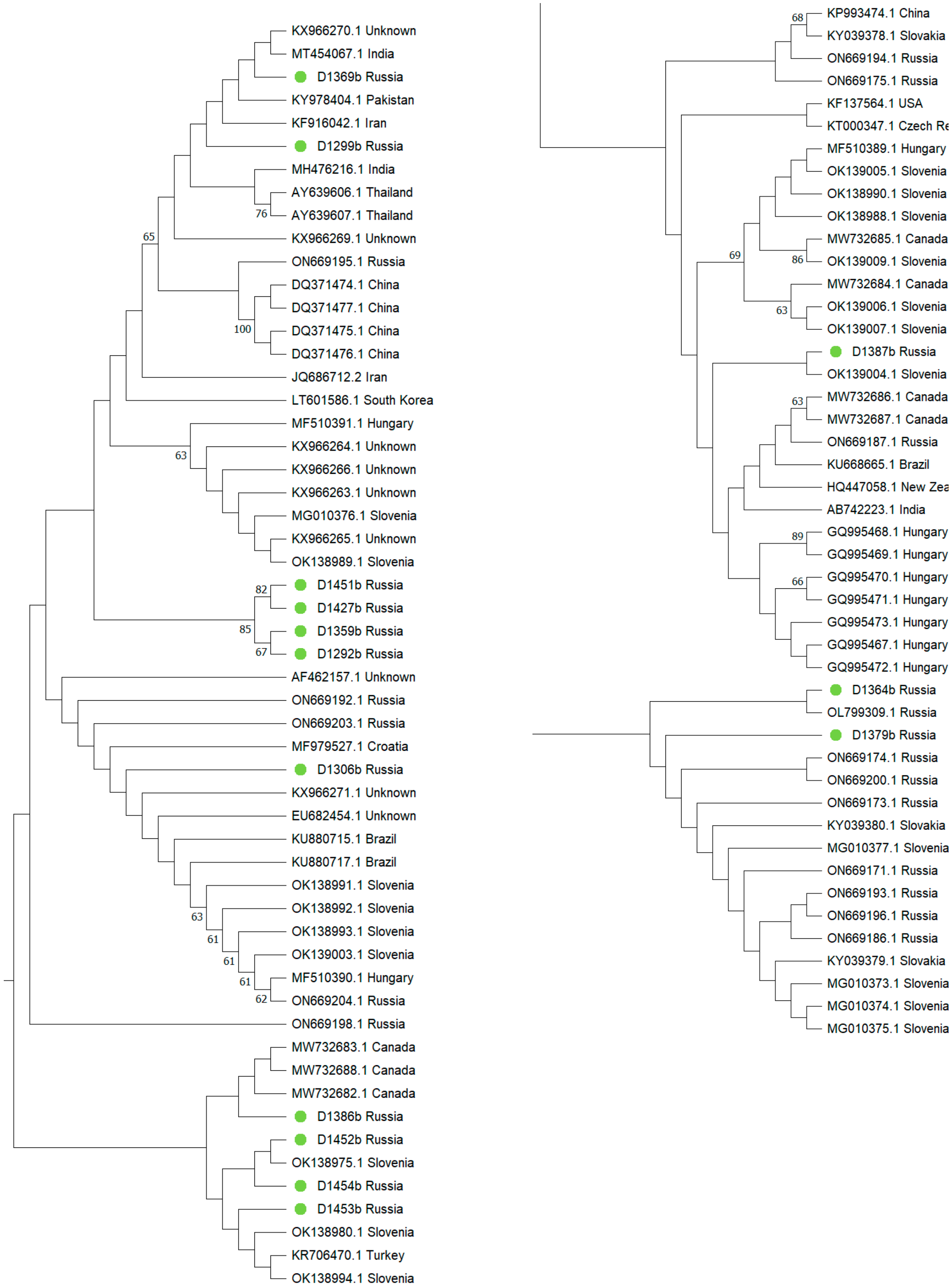
KX966270.1 Unknown
MT454067.1 India
D1369b Russia
KY978404.1 Pakistan
KF916042.1 Iran
D1299b Russia
MH476216.1 India
AY639606.1 Thailand
AY639607.1 Thailand
KX966269.1 Unknown
ON669195.1 Russia
DQ371474.1 China
DQ371477.1 China
DQ371475.1 China
DQ371476.1 China
JQ686712.2 Iran
LT601586.1 South Korea
MF510391.1 Hungary
KX966264.1 Unknown
KX966266.1 Unknown
KX966263.1 Unknown
MG010376.1 Slovenia
KX966265.1 Unknown
OK138989.1 Slovenia
D1451b Russia
D1427b Russia
D1359b Russia
D1292b Russia
AF462157.1 Unknown
ON669192.1 Russia
ON669203.1 Russia
MF979527.1 Croatia
D1306b Russia
KX966271.1 Unknown
EU682454.1 Unknown
KU880715.1 Brazil
KU880717.1 Brazil
OK138991.1 Slovenia
OK138992.1 Slovenia
OK138993.1 Slovenia
OK139003.1 Slovenia
MF510390.1 Hungary
ON669204.1 Russia
ON669198.1 Russia
MW732683.1 Canada
MW732688.1 Canada
MW732682.1 Canada
D1386b Russia
D1452b Russia
OK138975.1 Slovenia
D1454b Russia
D1453b Russia
OK138980.1 Slovenia
KR706470.1 Turkey
OK138994.1 Slovenia
KP993474.1 China
KY039378.1 Slovakia
ON669194.1 Russia
ON669175.1 Russia
KF137564.1 USA
KT000347.1 Czech Re
MF510389.1 Hungary
OK139005.1 Slovenia
OK138990.1 Slovenia
OK138988.1 Slovenia
MW732685.1 Canada
OK139009.1 Slovenia
MW732684.1 Canada
OK139006.1 Slovenia
OK139007.1 Slovenia
D1387b Russia
OK139004.1 Slovenia
MW732686.1 Canada
MW732687.1 Canada
ON669187.1 Russia
KU668665.1 Brazil
HQ447058.1 New Zea
AB742223.1 India
GQ995468.1 Hungary
GQ995469.1 Hungary
GQ995470.1 Hungary
GQ995471.1 Hungary
GQ995473.1 Hungary
GQ995467.1 Hungary
GQ995472.1 Hungary
D1364b Russia
OL799309.1 Russia
D1379b Russia
ON669174.1 Russia
ON669200.1 Russia
ON669173.1 Russia
KY039380.1 Slovakia
MG010377.1 Slovenia
ON669171.1 Russia
ON669193.1 Russia
ON669196.1 Russia
ON669186.1 Russia
KY039379.1 Slovakia
MG010373.1 Slovenia
MG010374.1 Slovenia
MG010375.1 Slovenia

(a) **(b)**

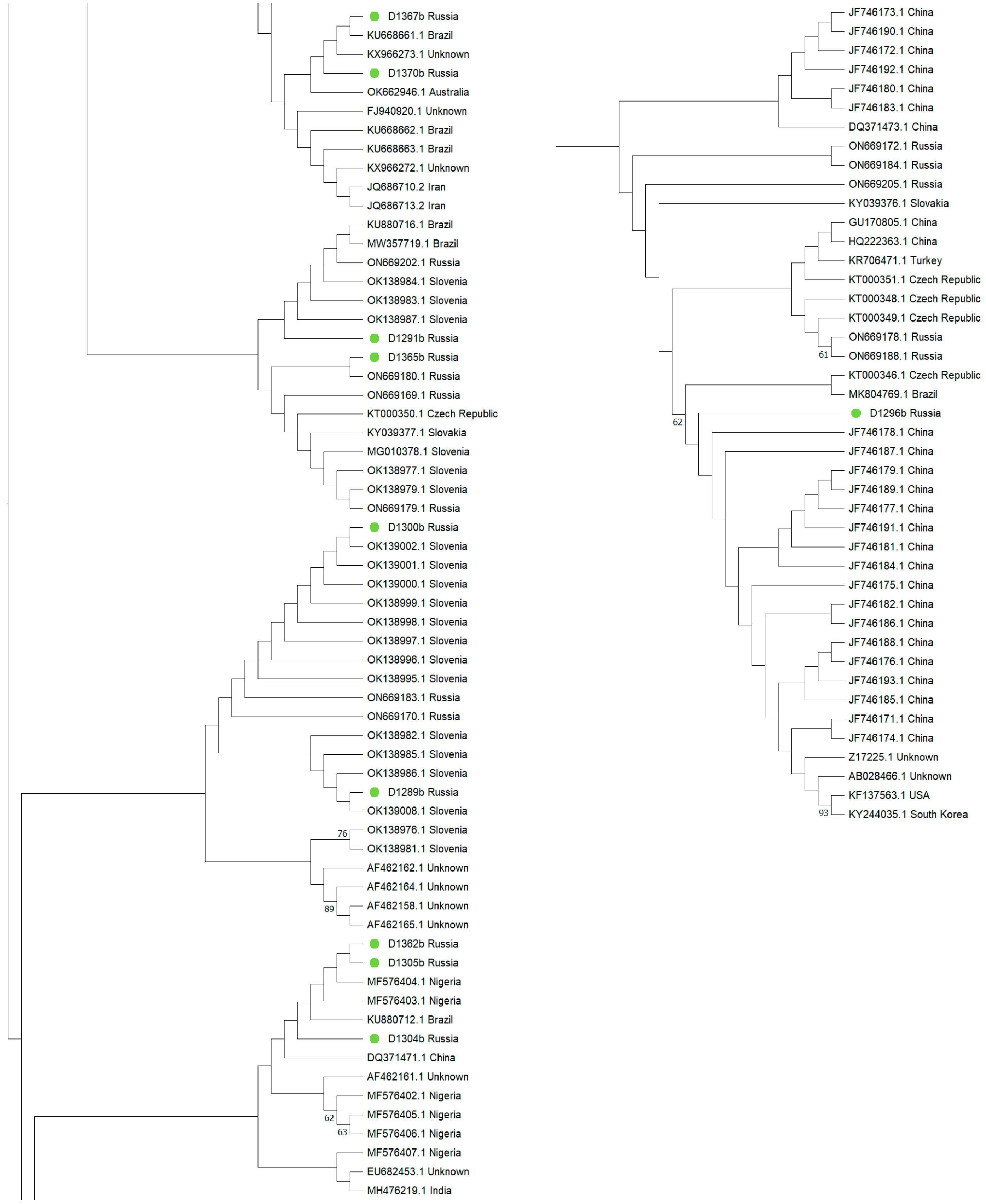

(c) (d)

Supplementary Figure S15 (a-d). Neighbor-joining tree showing the distribution of complete genome nucleotide sequences of Russian grapevine yellow speckle viroid 1 (GYSVd-1) isolates (●) and world isolates. Bootstrap values >60% (1000 bootstrap replicates) are shown.

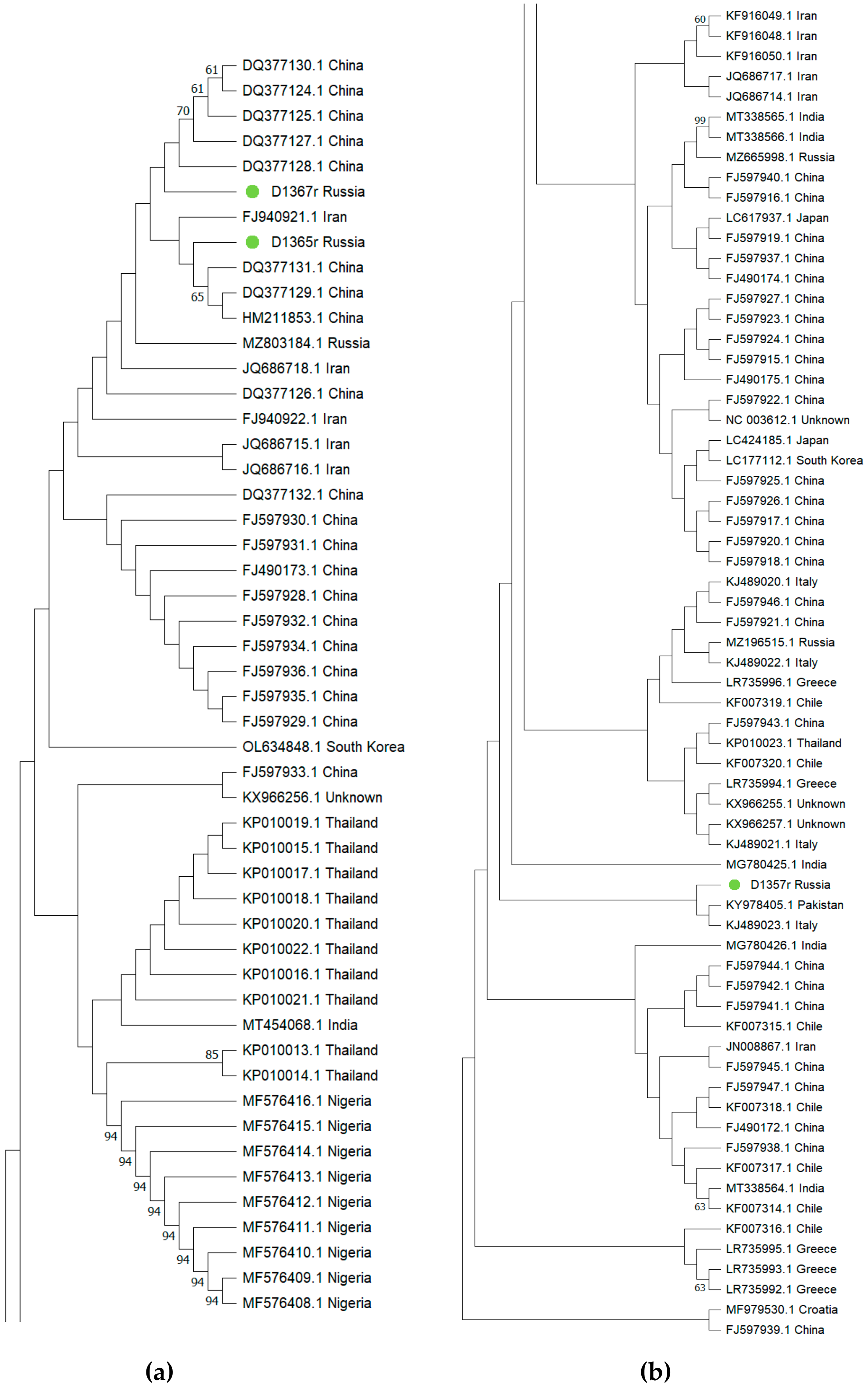

Supplementary Figure S16 (a, b). Neighbor-joining tree showing the distribution of complete genome nucleotide sequences of Russian grapevine yellow speckle viroid 2 (GYSVd-2) isolates (●) and world isolates. Bootstrap values >60% (1000 bootstrap replicates) are shown.

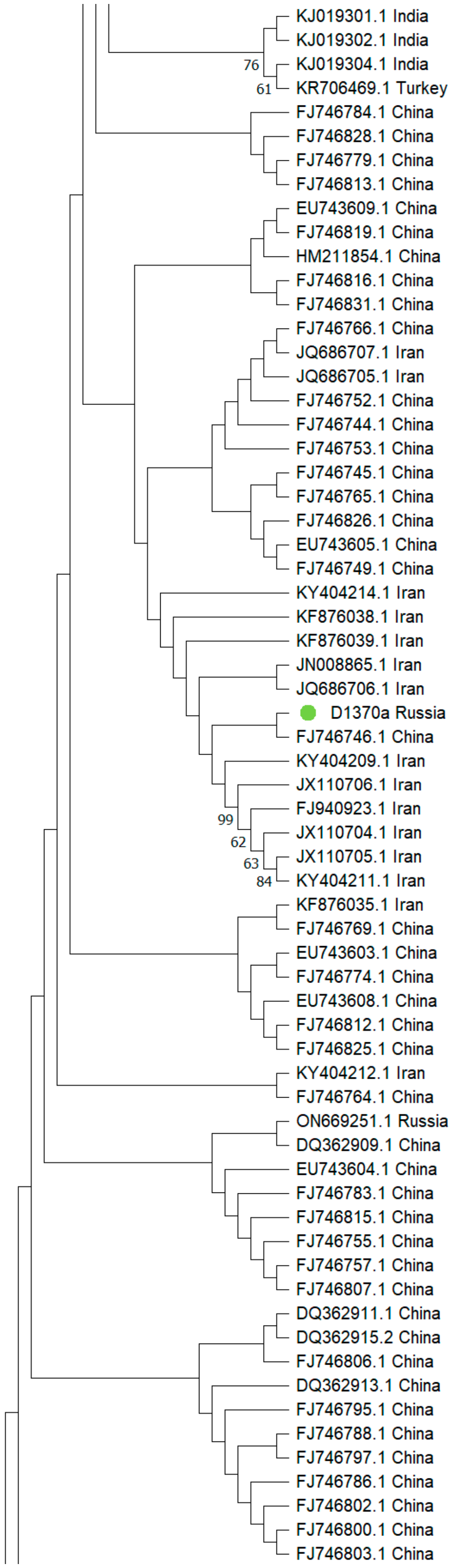

Supplementary Figure S17. Neighbor-joining tree showing the distribution of complete genome nucleotide sequences of Russian Australian grapevine viroid (AGVd) isolate (●) and world isolates. Bootstrap values >60% (1000 bootstrap replicates) are shown.
